# Genetic variation in GABRB3 is associated with Asperger syndrome and multiple endophenotypes relevant to autism

**DOI:** 10.1186/2040-2392-4-48

**Published:** 2013-12-09

**Authors:** Varun Warrier, Simon Baron-Cohen, Bhismadev Chakrabarti

**Affiliations:** 1 Department of Psychiatry, Autism Research Centre, University of Cambridge, Cambridgeshire, UK; 2 CLASS Clinic, Cambridgeshire and Peterborough NHS Foundation Trust (CPFT), Cambridgeshire, UK; 3 Centre for Integrative Neuroscience and Neurodynamics, School of Psychology and Clinical Language Sciences, University of Reading, Reading, UK

**Keywords:** Asperger syndrome, Autism spectrum conditions, Empathy, Embedded Figures Test, Mental Rotation Test, GABA receptor

## Abstract

**Background:**

Autism spectrum conditions (ASC) are associated with deficits in social interaction and communication, alongside repetitive, restricted, and stereotyped behavior. ASC is highly heritable. The gamma-aminobutyric acid (GABA)-ergic system has been associated consistently with atypicalities in autism, in both genetic association and expression studies. A key component of the GABA-ergic system is encoded by the *GABRB3* gene, which has been previously implicated both in ASC and in individual differences in empathy.

**Methods:**

In this study, 45 genotyped single nucleotide polymorphisms (SNPs) within *GABRB3* were tested for association with Asperger syndrome (AS), and related quantitative traits measured through the following tests: the Empathy Quotient (EQ), the Autism Spectrum Quotient (AQ), the Systemizing Quotient-Revised (SQ-R), the Embedded Figures Test (EFT), the Reading the Mind in the Eyes Test (RMET), and the Mental Rotation Test (MRT). Two-loci, three-loci, four-loci haplotype analyses, and one seven-loci haplotype analysis were also performed in the AS case–control sample.

**Results:**

Three SNPs (rs7180158, rs7165604, rs12593579) were significantly associated with AS, and two SNPs (rs9806546, rs11636966) were significantly associated with EQ. Two SNP-SNP pairs, rs12438141-rs1035751 and rs12438141-rs7179514, showed significant association with variation in the EFT scores. One SNP-SNP pair, rs7174437-rs1863455, was significantly associated with variation in the MRT scores. Additionally, a few haplotypes, including a 19 kb genomic region that formed a linkage disequilibrium (LD) block in our sample and contained several nominally significant SNPs, were found to be significantly associated with AS.

**Conclusion:**

The current study confirms the role of *GABRB3* as an important candidate gene in both ASC and normative variation in related endophenotypes.

## Background

Autism spectrum conditions (ASC) are neurodevelopmental, and are characterized by impairments in social interaction and communication alongside unusually narrow interests and repetitive behavior [1]. ASC are highly heritable, although environmental and epigenetic factors also contribute [2,3], and they affect 1% of the population [4]. Previous genetic research has largely focused on those individuals who also have learning difficulties. Asperger syndrome (AS) is a subgroup of ASC, and individuals with AS have no speech or cognitive delays [5].

Although no single gene accounts for all of the variation in the autism spectrum, several lines of evidence points to a common theme of neural development across genetic studies of autism. Genes involved in early brain development (including genes involved in synapse formation and stabilization, and those involved in neurotransmission in these early neural circuits) have been associated consistently with autism [6-8]. The gamma-aminobutyric acid (GABA)-ergic system plays a crucial role in early neural development. Both genetic association and gene expression studies suggest a key role for the GABA-ergic system in autism [9-11]. Specifically, variation in the *GABRB3* gene in humans has been previously implicated in both empathy [12] and ASC [13-16]. The first study to associate variants in *GABRB3* with ASC identified a statistically significant association between the marker 155CA-2 and individuals with ASC, using a multiple transmission disequilibrium test on 140 families [17]. This was replicated in 80 autism families [13]. Copy number variations and other chromosomal abnormalities at the *GABRB3* locus have also been reported in individuals with ASC [14,18].

Variation in *GABRB3* is also associated with tactile sensitivity, which is atypical in some individuals with ASC [19]. Gabrb3 knockout mice exhibit deficits in social behavior and constitute a potential mouse model for autism [20]. *GABRB3* encodes the B3 subunit of the GABA_A_ receptor. GABA_A_ receptor is an ionotropic, ligand-gated receptor, which is part of the inhibitory synapses in the adult brain, and selectively conducts Cl^-^ ions. During development, GABRB3 is an important molecule for neuronal growth and differentiation, and mediates excitatory signaling [21,22].

In the present study we tested the importance of *GABRB3* as a candidate gene in AS and in six potential endophenotypic measures of the autistic spectrum: three self-report measures (autistic traits, empathy, systemizing) and three performance measures (emotion recognition, attention to detail, and spatial processing).

The self-report measures used were as follows. The Autism Spectrum Quotient (AQ) is a measure of autistic traits. An AQ score above 32 is a predictor of ASC [23], and AQ scores show significant heritability in a general population sample [24]. Empathy was measured using the Empathy Quotient (EQ) [25]. On average, individuals with a diagnosis of AS will score significantly lower than controls on the EQ [25]. Several lines of research have suggested that individuals with ASC have difficulties with empathy, especially cognitive empathy [26]. Empathy in humans has a partly genetic basis [27,28]. Systemizing was measured using the Systemizing Quotient-Revised (SQ-R), in which individuals with AS score higher than controls [27,28]. Family genetic studies suggest that systemizing is part of the broader autistic phenotype in first-degree relatives of individuals with autism [29,30]. Although largely independent of each other, the EQ and the SQ-R predict scores on the AQ [31].

The performance measures were as follows. Complex emotion recognition ability was measured through the Reading the Mind in the Eyes test (RMET). This is an adult test of social sensitivity/cognitive empathy, in which individuals are shown photos of the eye region, and required to choose which word best represents what the person in the photograph is feeling or thinking [32]. On average, people with ASC score significantly lower than controls, and parents and siblings of people with ASC also show mild deficits in this test. Performance is normally distributed in the population, and scores on this test show a high degree of familiality [33]. Attention to detail was measured using the Embedded Figures Test (EFT), in which participants are asked to locate a target shape embedded within a complex pattern [34]. Individuals with ASC score above average [34], and scores on the EFT are also associated with high familiality [35]. Spatial processing was measured using the Mental Rotation Test (MRT), in which individuals are asked to process rotated pairs of letters and identify if the letter-pairs are identical to or mirror images of each other. Individuals with ASC perform better than controls on the MRT [36], and there is some evidence to suggest heritability of mental rotation ability [37].

To our knowledge, this is only the second study investigating the role of *GABRB3* specifically in AS, and variations in EQ, AQ, EFT, and RMET scores. The first study (also from our group) found a genetic association between a variant in *GABRB3* and empathy [12]. In the current study, we aimed to replicate and extend the findings of the first study in a new sample of volunteers. We hypothesized an association between the variants in *GABRB*3 with both AS and associated quantitative traits. Two separate experiments were conducted to test the role of *GABRB3* in adults with AS. The first tested if any of 45 single nucleotide polymorphisms (SNPs) showed an association with a diagnosis of AS, using a case–control design. The second experiment tested for association of these SNPs with any of the quantitative endophenotypic measures described above, in the control population.

## Methods

### Ethics approval

The study was approved by the Cambridge Psychology Research Ethics Committee and by the NHS Research Ethics Committee (UK). All participants provided written consent to take part in this study. All research adhered to the Declaration of Helsinki.

### Participants

All individuals involved in the current study were of Caucasian ancestry. Individuals free of any psychiatric or neurological disorders were recruited as controls by advertisements.

In total, 530 individuals participated in the case–control association study for AS. Of these, 412 (185 male, 227 female) individuals with an AQ score below 24 formed the controls, and 118 individuals with a DSM-IV (*Diagnostic and Statistical Manual of Mental Disorders, Fourth Edition)* or ICD-10 (*International Classification of Diseases, Tenth Revision*)*–*diagnosis of AS (74 male, 44 female) formed the cases. All cases were recruited through our online database, and were diagnosed with AS by independent clinicians (psychiatrists or clinical psychologists only) from established clinics. The mean AQ score of the cases was 35.6 ± 8.9. This is very close to the previously reported mean of 35.6 ± 6.63 for people with AS [21]. The mean AQ score in the general population is 16.4, with a standard deviation of 6.3 [38]. Taking this into consideration, we selected only individuals with an AQ score of below 24 as controls to ensure a balanced representation of individuals from two ends of the autistic trait continuum.

All the quantitative trait studies were conducted only in participants without a clinical diagnosis of AS. Individuals were invited to participate in online assessment of all six measures investigated in the study. In total, 412 individuals participated in the association study for the AQ, 413 for the EQ, and 414 for the SQ-R. The same individuals who were the controls in the case–control study participated in the association study for AQ. Additionally, 245 individuals participated in the association study for the MRT, REMT, and EFT.

Phenotypic scores for each measure and participant information are provided in Table 1.

**Table 1:** Phenotype measures and participant information

**Participants**	**Case–control**		**AQ**^**a**^	**EQ**	**SQ-R**	**RMET**^**b**^	**EFT**^**b**^	**MRT**^**b**^
	**Cases**	**Controls**^**a**^						
Numbers								
Total	118	412	412	413	414	245	245	245
Male	74	185	185	209	210	122	122	122
Female	44	227	227	204	204	123	123	123
Trait score, mean ± SD								
Overall	35.6 ± 8.9^c^	14.9 ± 5.0^c^	14.9 ± 5.0	41.5 ± 12.3	60.3 ± 20.6	27.1 ± 3.2	11.6 ± 1	16.8 ± 2.6
Male	35.1 ± 8.7^c^	16.0 ± 4.4^c^	16.0 ± 4.4	36.8 ± 12.4	63.9 ± 20.0	27.06 ± 3.2	11.7 ± 0.6	17.4 ± 2.1
Female	36.6 ± 8.8^c^	13.9 ± 5.1^c^	13.9 ± 5.1	45.6 ± 12.3	56.5 ± 20.4	27.1 ± 3.2	11.6 ± 1.2	16.1 ± 2.9
Range	7 to 50	2 to 23	2 to 23	2 to 24	11 to 139	17 to 34	2 to 12	7 to 20

*Abbreviations*: AQ, Autism Spectrum Quotient; EFT, Embedded Figures Test; EQ, Empathy Quotient; MRT, Mental Rotation Test; RMET, Reading the Mind in the Eyes Test; SQ-R, Systemizing Quotient, Revised.

^a^The same individuals participated in the case–control and AQ quantitative trait studies.

^b^The same individuals participated in REMT, EFT, and MRT.

^c^Trait scores for case–control study are the Adult Autism Spectrum Quotient scores.

### SNP selection

In total, 45 SNPs were selected to provide maximal coverage through linkage disequilibrium (LD) of the *GABRB3* gene region on chromosome 15 from 26802824 bp to 27200217 bp (GRCH37.p10 Primary Assembly, NCBI). SNPs were chosen to ensure that inter-SNP distance was less than 10 kb. The choice of SNPs was constrained by the availability of the ABI TaqMan assays that were used for genotyping. Nine SNPs (rs12437672, rs1432007, rs12905535, rs737098, rs2114485, rs1426217, rs890317, rs7171512, rs8026392) are TagSNPs as indicated by the HapMap genome browser, release 27. Although the SNPs covered most of the genetic region, they did not completely flank the gene. Of the 45 SNPs, rs3212331 (Chr15: 27014769) is the most upstream, and rs2114485 is the most downstream (Chr15: 26802824) in the orientation of the gene. For the selected SNPs, the minor allele frequency (MAF) was above 0.05 in the CEU (Utah Residents with Northern and Western European Ancestry) population as calculated from the dbSNP database (http://www.ncbi.nlm.nih.gov/projects/SNP/). DNA was extracted from buccal samples, anonymized, and genotyped using the same protocol described in our first study [12]. No SNPs deviated significantly from Hardy-Weinberg equilibrium. Total genotyping rate across all the experiments was greater than 98%.

### Association tests

The two experiments were as follows.

#### Case–control association study for AS

SNP association tests were conducted for all SNPs on all cases with AS (n = 118), and controls (n = 412, with an AQ score below 24). SNP-SNP interaction tests were then carried out on this sample. In total, 990 possible SNP-SNP combinations (45 × 45) were tested for association using logistic regression, and significant SNPs were additionally checked for LD between them. Interactions were reported to be significant if the SNP pairs were not in LD.

#### Quantitative trait association study for AS

Quantitative trait association studies were performed for the AQ, SQ-R, EQ, ET, EFT and MRT. SNP association tests on each of these quantitative traits were conducted in controls only. The number of participants in each experiment differed (Table 1). SNP-SNP interaction tests were tested for association with each of the six quantitative traits, for a total of 990 SNP combinations, and modeled using linear regression. As in the previous experiment, interactions were reported to be significant if the SNP pairs were not in LD.

### Statistical analysis

All statistical analyses were performed using Plink version 1.07 (http://pngu.mgh.harvard.edu/~purcell/plink/) [39]. Bonferroni correction was implemented to correct for multiple SNPs and phenotypes tested. A threshold of significance (α) was calculated after both the corrections. *P*-values were reported as significant if they were below the α threshold.

The effective number of independent SNPs was estimated using SNPSpD (http://genepi.qimr.edu.au/general/daleN/SNPSpD/) [40]. For the SNP association studies the total number of independent loci (after taking into account the LD between SNPs) and the new α value was determined for each test using SNPSpD. On average, the total number of independent loci was calculated to be 30.5, although this varied marginally between tests (because of small differences in the number of individuals who completed each quantitative measure). For SNP-SNP interaction, a total of 990 possible SNP-SNP combinations was tested. However, SNP-SNP interaction can be calculated accurately only for independent loci. The total number of independent SNP-SNP combinations was thus determined to be 465 (31 × 30)/2). The Bonferroni corrected α was set to 0.000108.

Bonferroni correction was also used to correct for the multiple phenotypes tested. Owing to differences in samples for the phenotypes tested the experiments were broadly divided into three groups for correction.

Group 1 was the case–control association test for AS, which had the largest sample size and was distinct because of inclusion of cases (Experiment 1). In this sample, only one phenotype (that is, clinical diagnosis) was tested, and hence correction for multiple phenotypes was not implemented.

Experiment 2 consisted of two largely distinct samples. The first of these samples, group 2, comprised the AQ, EQ and SQ-R, as more than three-quarters of the participants had participated in two or more of these tests. However, as all three phenotypes had a high degree of correlation between them, these were not treated as truly independent observations. Because Bonferroni correction tends to be over-corrective when traits are correlated, we did not use this for multiple phenotypes in this group.

The second sample within experiment 2, group 3, comprised the three performance-based tests (the RMET, EFT, and MRT) and was a subsample of group 2.

In total, 245 individuals took part in all three tests. There was little correlation between the phenotype scores for these tests in our sample. Bonferroni correction was implemented for this group. The α value for SNP association tests and SNP-SNP interaction tests derived from SNPSpD was divided by 3 to get the final α value. For SNP-SNP interaction tests on the performance-based tests, the final α value was 0.000036 (or 0.000108 divided by 3).

### Haplotype analysis

Haplotype analysis was performed for case–control AS dataset using Plink. Haplotypes were phased using the E-M algorithm, and global or omnibus two-loci, three-loci, and four-loci haplotype analyses were performed using logistic regression on Plink. Additionally, one large LD block, which is 19 kb long from rs7174437 to rs7180158, was considered for haplotype analysis. There are nine SNPs located in this region, including all three significant SNPs and three nominally significant SNPs for the case–control AS association study. *P*-values were corrected for family-wise error rates using permutation correction with 50,000 permutations, and the α value for significance was kept at 0.05.

### LD analyses and SNP annotation

LD values between SNPs of interest in the HapMap CEU population data were calculated using SNAP (http://www.broadinstitute.org/mpg/snap/) [41]. LD values between SNPs in our sample were calculated using Plink. LD values have been reported using R^2^ rather than D′ as R^2^ takes into account allele frequencies in populations. LD plots for the sample studied were created using Haploview [42].

Integrated putative functional scores for the SNPs tested were obtained using both the F-SNP server (http://compbio.cs.queensu.ca/F-SNP) [43] and FastSNP (http://fastsnp.ibms.sinica.edu.tw/pages/input_CandidateGeneSearch.jsp) [44]. SNPnexus (http://www.snp-nexus.org/) [45] was used to investigate SNP conservation and structural variants in the locus investigated. Haploreg (http://www.broadinstitute.org/mammals/haploreg/) [46] was used to check for histone markers, conservation scores, and DNAse hypersensitivity sites for the SNP of interest and other SNPs in high LD (R^2^ > 0.8). This was also manually cross-checked using the UCSC Genome Browser (http://genome.ucsc.edu/) [47]. Finally, the Genetic Association Database (http://geneticassociationdb.nih.gov/) [48] was used to check for reports of any previous associations for the significant SNPs.

## Results

### SNP associations

#### Case–control association

**Figure 1:**
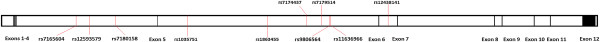
**Schematic diagram of *GABRB3 *showing significant single nucleotide polymorphisms (SNPs).** Black lines represent exons. Red lines represent the significant SNPs. There are 12 exons in *GABRB3*, which are shown.

**Figure 2:**
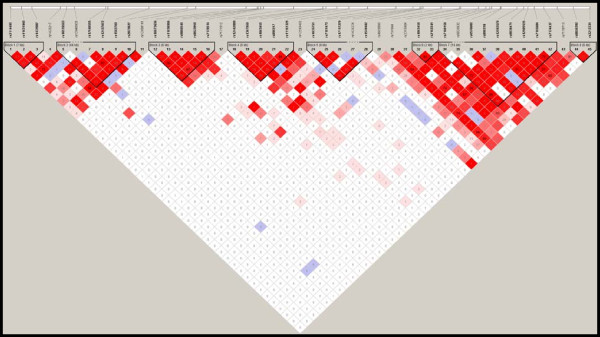
**Linkage disequilibrium (LD) structure of *GABRB3 *for the Asperger syndrome (AS) case–control dataset.** LD structure of the region investigated in the AS case–control dataset (412 controls and 118 cases), as plotted on Haploview is shown. In this graph, 45 single nucleotide polymorphisms (SNPs) that were genotyped have been plotted. They fall into eight LD blocks.

Ten SNPs were nominally associated with AS (Table 2). Of these, rs7180158, rs7165604, and rs12593579 survived Bonferroni correction (Table 3). All three SNPs are located in the intron 4 of the *GABRB3* gene (Figure 1). In our sample, all three SNPs were found to be a part of an LD block as calculated by Haploview (Figure 2). Nine SNPs form the LD block. Three of the SNPS (rs7180158, rs7165604, and rs12593579) were significant after Bonferroni correction and another three of these SNPs (rs12905535, rs7174437, rs6576602) were nominally significant in the AS association study. For the LD structure for *GABRB3* in the HapMap CEU population, see Additional file 1. There were no significant SNP-SNP interactions for the case–control analysis (see Additional file 2).

**Table 2:** GABRB3 SNP association results for AS and the six quantitative endophenotypes investigated

**SNP**	**SNP position**	**AS case–control**		***P *** **value**					
		**OR**	**P value**	**AQ**	**EQ**	**SQ-R**	**EFT**	**REMT**	**MRT**
rs2114485	26802824	0.9369	0.704	0.6105	0.6514	0.6369	0.6719	0.1518	0.9454
rs11631940	26806814	0.8902	0.5088	0.6759	0.8435	0.1997	0.8645	0.8271	0.4787
rs1432007	26810689	1.127	0.4205	0.7813	0.6526	0.5193	0.7481	0.2376	0.7725
rs1426217	26821125	1.082	0.5975	0.439	0.4008	0.5922	0.5827	0.6484	0.5826
rs10519563	26823404	1.096	0.6204	0.8042	0.1096	0.1448	0.3941	0.9824	0.2969
rs12440905	26826281	1.584	0.3943	0.9656	0.2318	0.9096	0.5663	0.6207	0.5445
rs17646555	26837651	1.089	0.749	0.7359	0.0351	0.7584	0.6143	0.2616	0.1865
rs12437672	26838405	1.016	0.9246	0.6538	0.3636	0.4328	0.8751	0.675	0.4629
rs1582760	26853487	1.114	0.6609	0.902	0.2335	0.7375	0.4951	0.4093	0.3209
rs2873027	26867409	0.9804	0.8947	0.4164	0.7438	0.4436	0.3401	0.996	0.7369
rs12438141	26870602	1.094	0.6625	0.2593	0.05641	0.7694	0.04683	0.1429	0.004928
rs10873636	26888978	0.8377	0.2985	0.01893^a^	0.01765^a^	0.04946^a^	0.8912	0.05818	0.02446
rs11636966	26893028	0.9915	0.9547	0.06219	0.000594^a^	0.8307	0.7713	0.5205	0.7296
rs9806546	26893386	0.7572	0.188	0.06427	0.001469	0.009834^a^	0.6132	0.4449	0.3145
rs8023959	26894305	1.034	0.887	0.1065	0.7408	0.8473	0.3852	0.05979	0.06356
rs7179514	26895324	1.028	0.8525	0.107	0.002318^a^	0.5474	0.3509	0.5521	0.1739
rs7171512	26906345	0.9611	0.7976	0.3705	0.01302^a^	0.3096	0.7664	0.1414	0.9829
rs12442889	26917100	1.041	0.8066	0.3348	0.2381	0.3188	0.8606	0.6945	0.1025
rs1367959	26917158	1.178	0.315	0.7905	0.001742^a^	0.1215	0.02745	0.2206	0.05204
rs1863455	26918169	0.8474	0.4769	0.2206	0.5058	0.4483	0.3591	0.005442	0.8047
rs890317	26922201	0.8293	0.2741	0.9942	0.1927	0.9031	0.09281	0.887	0.8351
rs11161329	26926710	0.8283	0.2203	0.5383	0.07182	0.6331	0.02263	0.07253	0.7137
rs12593482	26933545	1.119	0.7621	0.7125	0.04769^a^	0.2482	0.6505	0.9041	0.6601
rs1035751	26943796	0.8033	0.4228	0.531	0.8884	0.9924	0.1161	0.9053	0.4785
rs7181473	26950087	1.018	0.9072	0.4267	0.5739	0.9399	0.7201	0.8816	0.6508
rs17117279	26952672	0.8777	0.5218	0.3016	0.09375	0.6948	0.1173	0.2397	0.4522
rs1426224	26953091	0.984	0.9632	0.3725	0.1801	0.2826	0.5186	0.1248	0.4864
rs1549482	26953764	1.073	0.7015	0.9659	0.6341	0.6026	0.072	0.4522	0.3501
rs4906896	26960426	1.354	0.04067^a^	0.001682^a^	0.01946^a^	0.8831	0.05366	0.03463^a^	0.126
rs737098	26967170	0.7071	0.04886^a^	0.1189	0.1006	0.03769^a^	0.9156	0.5102	0.6054
rs2315904	26967522	0.8293	0.4539	0.4212	0.5888	0.08328	0.1348	0.3303	0.1788
rs1863456	26974032	1.006	0.9703	0.116	0.06622	0.7006	0.2814	0.6925	0.271
rs2162241	26976782	0.7075	0.02707^a^	0.2351	0.1778	0.2413	0.5519	0.5273	0.8923
rs7180158^b^	26978238	2.15	0.000358^a^	0.4416	0.2842	0.01907^a^	0.3974	0.7486	0.124
rs8026932^b^	26978906	1.263	0.5769	0.006922^a^	0.9863	0.7881	0.6083	0.1999	0.9726
rs6576602^b^	26982080	1.46	0.03347^a^	0.189	0.3375	0.00998^a^	0.05174	0.4184	0.01776^a^
rs890318^b^	26982378	0.745	0.2896	0.2458	0.8865	0.3377	0.08322	0.4733	0.1141
rs12593579^b^	26988132	1.96	0.000668^a^	0.5066	0.335	0.03631^a^	0.3711	0.74	0.2056
rs8038471^b^	26990651	1.154	0.3372	0.08613	0.01078^a^	0.9937	0.3891	0.8799	0.6707
rs12905535^b^	26992166	0.6169	0.005747^a^	0.08597	0.03931^a^	0.04997^a^	0.283	0.4318	0.07415
rs7165604^b^	26994456	2.452	0.000451^a^	0.1189	0.1297	0.1982	0.278	0.8782	0.09043
rs7174437^b^	26997923	2.022	0.01567^a^	0.1185	0.02142^a^	0.5118	0.4765	0.8321	0.4211
rs7178713	27002886	0.6789	0.01877^a^	0.4842	0.1292	0.1468	0.5308	0.1201	0.1008
rs8026392	27008723	0.8825	0.4244	0.05513	0.02792^a^	0.4595	0.4105	0.6825	0.3217
rs3212331	27014769	0.896	0.53	0.6579	0.7116	0.6821	0.2821	0.8648	0.5415

*Abbreviations*: *AQ* Autism Spectrum Quotient, *EFT* Embedded Figures Test, *EQ* Empathy Quotient, *MRT* Mental Rotation Test, *RMET* Reading the Mind in the Eyes Test, *SQ-R* Systemizing Quotient, Revised, *SNP* single nucleotide polymorphism.

^a^Nominally significant *P*-values (*P* < 0.05).

^b^SNPs found in haplotype block 7 (as referred to in Figure 2).

**Table 3:** Significant haplotypes for case–control (Asperger syndrome)

**No of SNPs**	**SNP haplotype**	**FWER corrected *P*-value**
2	rs1863456-rs2162241	0.03284
2	rs2162241-rs7180158	0.03882
2	rs7180158-rs8026932	0.0304
2	rs6576602-rs890318	0.03576
2	rs890318-rs8038471	0.021
2	rs12593579-rs8038471	0.00582
2	rs12905535-rs7165604	0.01768
3	rs4906896-rs737098-rs2315904	0.02002
3	rs1863456-rs2162241-rs7180158	0.02438
3	rs8026932-rs6576602-rs890318	0.0209
3	rs890318-rs12593579-rs8038471	0.0145
3	rs12593579-rs8038471-rs12905535	0.01192
3	rs8038471-rs12905535-rs7165604	0.03052
4	rs7180158-rs8026932-rs6576602-rs890318	0.03832
4	rs8026932-rs6576602-rs890318-rs12593579	0.04774
4	rs6576602-rs890318-rs12593579-rs8038471	0.02428
4	rs890318-rs12593579-rs8038471-rs12905535	0.01002
4	rs12593579-rs8038471-rs12905535-rs7165604	0.0454
9	rs7180158-rs8026932-rs6576602-rs890318-rs12593579-rs8038471-rs12905535-rs7165604-rs7174437	0.00534

*Abbreviations*: *FWER* family-wise error rate, *SNP* single nucleotide polymorphism.

#### Quantitative trait associations

Two SNPs were nominally associated with the RMET, seven were nominally associated with the SQ-R, and 13 were nominally associated with the EQ, while three SNPs each were nominally associated with the AQ, EFT, and MRT (Table 2). Two SNPs, rs11636966 and rs9806546, survived Bonferroni correction for the EQ (see Table 4); both of these SNPs are located in intron 5 of the *GABRB3* gene (Figure 1). No SNP was nominally significant across all the six measures. Two SNP-SNP interactions were found to be significantly associated with the EFT after Bonferroni correction (Table 4). These were: rs12438141-rs1035751 (R^2^ = 0.017) and rs12438141-rs7179514 (R^2^ = 0.001) (Table 4, Figure 1). One SNP-SNP interaction, rs7174437-rs1863455, was found to be significant for the MRT (R^2^ = 0.003) (Table 4, Figure 1). Neither of the SNPs was nominally significant in the SNP association study for the MRT. There were no significant SNP-SNP interactions for any of the other traits tested (see Additional file 3).

**Table 4:** Significant SNPs and SNP-SNP pairs

**SNP or SNP-SNP interaction**	**Phenotype**	**P values (uncorrected)**^**a**^	**Minor allele/major allele**^**b**^	**MAF (HapMap CEPH)**^**c**^	**Frequency of minor allele**		**χ**^**2**^
					**Cases**	**Controls**	
rs7180158	AS	3.58E-04	A/G	A = 0.24	0.1624	0.08272	12.74
rs7165604	AS	4.51E-04	C/T	C = 0.17	0.1174	0.05145	12.31
rs12593579	AS	6.68E-04	C/A	C = 0.21	0.194	0.1093	11.58
rs11636966	EQ	5.94E-04	T/C	T = 0.22	NA	NA	NA
rs9806546	EQ	1.47E-03	A/G	A = 0.40	NA	NA	NA
rs12438141-rs1035751	EFT	7.59E-06	C/T - A/G	T = 0.13, G = 0.06	NA	NA	NA
rs12438141-rs7179514	EFT	5.21E-06	C/T - C/G	T = 0.13, G = 0.31	NA	NA	NA
rs7174437-rs1863455	MRT	1.58E-05	C/T - C/T	C = 0.12, C = 0.08	NA	NA	NA

*Abbreviations*: AQ, Autism Spectrum Quotient; AS, Asperger syndrome; EFT, Embedded Figures Test; EQ, Empathy Quotient; MRT, Mental Rotation Test; NA, not applicable; SNP, single nucleotide polymorphism.

^a^Corrected P-value threshold is 3.6 × 10^-05^.

^b^Alleles for SNP-SNP pairs have been provided in the order they are written.

^c^MAF for SNP-SNP pairs have been provided in the order they are written.

#### Haplotype analysis

Seven two-loci haplotypes, six three-loci haplotypes, five four-loci haplotypes, and one nine-loci haplotype, which spanned a 19 kb entire LD block (rs7180158-rs7174437; Chr15:26978238–26997923), were significant after permutation correction (Table 3; see Additional file 4). All the haplotypes except two two-loci haplotypes [rs1863456-rs2162241 (Chr15: 26974032–26976782) and rs2162241-rs7180158 (Chr15: 26976782–26978238)] and two three-loci haplotypes (rs4906896-rs737098-rs2315904 (Chr15: 26960426–26967522) and rs1863456-rs2162241-rs7180158 (Chr15: 26974032–26978238)) are part of the 19 kb region. The four haplotypes not a part of this region lie immediately upstream (see Figure 2) The omnibus nine-loci haplotype had a corrected *P*-value of 0.00534, indicating that the genomic region as a whole (Figure 2) is associated with AS in our sample.

## Discussion

In this study, we tested multiple common variants in *GABRB3* for association with AS and related endophenotypes in a general population sample. To our knowledge, this is the first study to incorporate such a comprehensive group of phenotypes (including clinical diagnosis, self-report questionnaires, and observational measures on both social and non-social aspects of autistic cognition). Nine SNPs were nominally associated with AS, of which three (rs7180158, rs7165604, rs12593579) retained their significance after Bonferroni correction. All three SNPs are located in a 19 kb genomic region that forms an LD block in our sample. The entire block was found to be associated with AS when tested for global haplotype association. For the EQ, thirteen SNPs were nominally significant, of which two (rs9806546, rs11636966) remained significant after Bonferroni correction. Two SNP-SNP interactions were significant for the EFT (rs12438141-rs1035751 and rs12438141-rs7179514) and one for the MRT (rs7174437-rs1863455) after Bonferroni correction. None of the SNPs was located in regions of common structural variations, CpG islands, or affected micro RNA binding sites, as calculated by SNPnexus and the UCSC Genome Browser.

The three SNPs that were significant in AS case–control studies are located in intron 4, within a region of 16,218 base pairs. In our sample, they were found to be a part of a shared 19 kb LD block, which is also associated with AS. This genomic region extends from rs7180158 (Chr15: 26978238, dbSNP) to rs7174437 (Chr15: 26997923, dbSNP) in intron 4, and is 19,905 bp downstream of exon 3 and 16,326 bp upstream of exon 4. When queried in FastSNP, rs12593579 was suggested to disrupt an enhancer region. Both rs7165604 and rs12593579 are found in putative transcription regulatory sites according to F-SNP. Further, according to Haploreg, these SNPs disrupt transcription factor binding sites either directly or through common variants in high LD. The two SNPs significantly associated with the EQ are located in intron 5, and are a mere 358 bp apart. However, they are not in significant LD either in the CEU population (R^2^: 0.182; see Additional file 1: Table S1) or in our sample (R^2^ = 0.157) (see Additional file 1: Figure S2). It is hence likely that the effect of the SNPs have been independently measured, although this does not exclude the possibility that a single non-tested variant situated close by is contributing to the effect seen at the two SNPs. Genotyping all the variants in this short region and testing them for association with the EQ should be undertaken in a future study. The most significantly associated SNP rs11636966 markedly alters an En-1_2 binding site (Haploreg). There is considerable evidence to suggest a role for proteins of the Engrailed family in early neurodevelopment and ASC [49-52]. Both these SNPs are also in high LD with several SNPs that putatively alter other transcription factor binding sites. A previous study from our laboratory [12] tested for association between two SNPs in *GABRB3* (rs2873027 and rs11161335) with both AS and the EQ. Neither of the two SNPs was significantly associated with AS, but rs2873027 was found to be associated with the EQ. In the current study, only rs2873027 was included in the 45 SNPs genotyped. rs2873027 was not significantly associated with the EQ in the current study.

Two SNP-SNP interactions were found to contribute to variation in the EFT scores. Three SNPs participate in these interactions and none of them is in significant LD with each other either in the CEU population sample or in our sample (see Additional file 1: Table S1; see Additional file 1: Figure S3). Two of these SNPs, rs7179514 and rs1035751, are located in intron 5, while rs12438141 is located in intron 6. According to both FastSNP and F-SNP, rs1035751 and rs12438141 alter transcription regulatory sites. All SNPs except rs1035751 disrupt regulatory motifs (Haploreg). A single SNP-SNP interaction, rs1863455-rs7174437, was significantly associated with the MRT. rs1863455 is located in intron 5 and rs7174437 in intron 4, and they are 79,754 bp apart. They are not in significant LD (R^2^ = 0.003) (see Additional file 1: Table S1) in the CEU population, and are not a part of an LD block in our sample (see Additional file 1: Figure S3). Both the SNPs disrupt regulatory motifs (FastSNP, F-SNP, and Haploreg). rs7174437 disrupts regulatory motifs of the Sox family of transcription factors (Haploreg), which play a pivotal role during development [53].

The putative functional annotation of the SNPs indicates that a number of the significantly associated SNPs alter *GABRB3* transcription during and after embryonic development either directly or via other variants that are in high LD. Of the ten SNPs found to be significant, three are in intron 4, six in intron 5, and one in intron 6. *GABRB3* has twelve exons (see Figure 1) and four transcript variants in NCBI. Transcript variants 1 and 2 are more common at a cellular level, but neither of them have exons 5 and 6. Transcript variant 3, which has been described only at the transcript level, starts from exon 5, whereas transcript variant 4, which has been described at the protein level, starts from exon 6. There are currently no studies describing the function of these transcripts. It is possible that the variants in introns 4 and 5 act to regulate levels of these alternate transcripts. Alternatively, the variants could also act as intronic regulatory elements to alter the levels of transcript variants 1 and 2. Cellular studies need to be performed to validate either of these hypotheses.

We did not replicate the exact SNP association reported by Chakrabarti *et al*. [12]. However, the inference in our earlier paper was at the gene level (that is, the statistical inference from the permutation analysis was that one or more SNPs in the gene were significantly associated with the phenotype, and not specifically for any SNP). The current study replicates the association of *GABRB3* with empathy at the gene level, and provides evidence for the association of *GABRB3* with AS, the EFT, and the MRT.

A limitation of this study is the moderate sample size. Replication of the results in a larger sample size is required to validate the findings. Although no SNP was significant across two or more traits, the experiments were not completely independent as several individuals participated in two or more experiments. Additionally, the low variance in the phenotypic scores of EFT must be taken into account when interpreting the results. To assess if the limited variance was restricted to the genotyped sample, we checked for EFT scores in a larger database. Both the mean EFT score and standard deviation were similar between the two datasets, indicating that the low variance in the EFT scores in our sample was not an artifact. Although none of the cases studied were formally tested for IQ, an essential feature for the clinical diagnosis of AS is that individuals diagnosed as having AS or high-functioning autism (as was the case for the ASC sample in our study) is that individuals have an IQ in the average range [54]. Average range is defined as two standard deviations either side of the mean, so this would mean an IQ of over 70. Further, it has been shown that IQ and autistic traits show limited genetic covariance [55]. It is therefore reasonable to infer that IQ differences are unlikely to confound the conclusions drawn from the current study. Finally, it is important to remember that *GABRB3* is not the sole gene that contributes to either AS or the traits tested, and that other genes could act either independently of or epistatically with *GABRB3* to contribute to these complex phenotypes.

## Conclusions

We report novel associations of SNPs in *GABRB3* with AS, and normative variation in empathy. We did not find any allelic association between SNPs in *GABRB3* and the five other endophenotypes tested. A large 19 kb LD block was also positively associated with AS in our sample. We also found three intragenic SNP-SNP interactions that are significantly associated with two of the endophenotypes. Two SNP-SNP interactions were associated with EFT, and one SNP-SNP interaction is associated with MRT. This is the first study to specifically test the association of such a large number of SNPs in *GABRB3* with AS, along with a comprehensive group of endophenotypes.

## Abbreviations

AQ: Autism spectrum quotient; AS: Asperger syndrome; ASC: Autism spectrum conditions; EFT: Embedded figures test; EQ: Empathy quotient; GABA: Gamma-aminobutyric acid; GABRB3: Gamma-aminobutyric acid receptor subunit beta-3; LD: Linkage disequilibrium; MRT: Mental rotation test; RMET: Reading the mind in the eyes test; SNP: Single nucleotide polymorphism; SQ-R: Systemizing quotient, revised.

## Competing interests

The authors declare they have no competing interests.

## Authors’ contributions

BC and SBC co-designed the study; VW and BC co-conducted the analysis, and SBC obtained funding for the study. All authors wrote the paper. All authors read and approved the final manuscript.

## Supplementary Material

Additional file 1 **Linkage disequilibrium (LD) information for the single nucleotide polymorphisms (SNPs) investigated.** A table presenting pairwise LD for the significant *GABRB*3 SNPs genotyped in the HapMap CEU population. Three LD plots for the region investigated in 1) the CEU population; 2) the cohort investigated for the Empathy Quotient (EQ); 3) the cohort investigated for the Embedded Figures Test (EFT), Mental Rotation Test (MRT) and Reading the Mind in the Eyes Test (RMET). Click here for file

Additional file 2 ***GABRB*** **3 SNP-SNP interaction results for case–control (AS).** AS, Asperger syndrome; SNP, Single nucleotide polymorphism. Click here for file

Additional file 3 ***GABRB3 *** **SNP-SNP interaction results (*P*-values) for the six quantitative traits investigated.** SNP, Single nucleotide polymorphism. Click here for file

Additional file 4 ***GABRB3 *** **case–control (AS) results for haplotype analysis.** AS, Asperger syndrome. Click here for file
