# Supplementary material for: Genetic variation in GABRB3 is associated with Asperger syndrome and multiple endophenotypes relevant to autism
Source: Mol Autism. 2013 Dec 9;4:48. doi: 10.1186/2040-2392-4-48 (PMC3903107; doi:10.1186/2040-2392-4-48)
Supplement: Additional file 1 — Linkage disequilibrium (LD) information for the single nucleotide polymorphisms (SNPs) investigated. A table presenting pairwise LD for the significant GABRB3 SNPs genotyped in the HapMap CEU population. Three LD plots for the region investigated in 1) the CEU population; 2) the cohort investigated for the Empathy Quotient (EQ); 3) the cohort investigated for the Embedded Figures Test (EFT), Mental Rotation Test (MRT) and Reading the Mind in the Eyes Test (RMET). [file 2040-2392-4-48-S1.docx]

**Additional File 4**

**LD information for the SNPs investigated**

**Table 1: Pairwise LD for the significant GABRB3 SNPs genotyped in the HapMap CEU population.**

|  | Trait | AS | AS | AS | EQ | EQ | EFT | EFT | EFT | MRT | MRT |
| --- | --- | --- | --- | --- | --- | --- | --- | --- | --- | --- | --- |
| Trait | SNP | rs7180158 | rs7165604 | rs12593579 | rs11636966 | rs9806546 | rs1035751 | rs12438141 | rs7179514 | rs7174437 | rs1863455 |
| AS | rs7180158 | 1 | 0.614 | 0.731 | 0.028 | 0.037 | 0.015 | 0.01 | 0.027 | 0.254 | 0.01 |
| AS | rs7165604 | 0.614 | 1 | 0.558 | 0.006 | 0.06 | 0.022 | 0.007 | 0.004 | 0.414 | 0.006 |
| AS | rs12593579 | 0.731 | 0.558 | 1 | 0.009 | 0.026 | 0.038 | 0.005 | 0.001 | 0.231 | 0.011 |
| EQ | rs11636966 | 0.028 | 0.006 | 0.009 | 1 | 0.182 | 0.06 | 0 | 0.679 | 0.027 | 0.003 |
| EQ | rs9806546 | 0.037 | 0.06 | 0.026 | 0.182 | 1 | 0.014 | 0.021 | 0.246 | 0 | 0.019 |
| EFT | rs1035751 | 0.015 | 0.022 | 0.038 | 0.06 | 0.014 | 1 | 0.017 | 0.068 | 0.051 | 0.016 |
| EFT | rs12438141 | 0.01 | 0.007 | 0.005 | 0 | 0.021 | 0.017 | 1 | 0.001 | 0.007 | 0.109 |
| EFT | rs7179514 | 0.027 | 0.004 | 0.001 | 0.679 | 0.246 | 0.068 | 0.001 | 1 | 0.02 | 0.001 |
| MRT | rs7174437 | 0.254 | 0.414 | 0.231 | 0.027 | 0 | 0.051 | 0.007 | 0.02 | 1 | 0.003 |
| MRT | rs1863455 | 0.01 | 0.006 | 0.011 | 0.003 | 0.019 | 0.016 | 0.109 | 0.001 | 0.003 | 1 |

R-sq LD values given.

**Figure 1: LD structure of GABRB3 for the CEU population.**

**
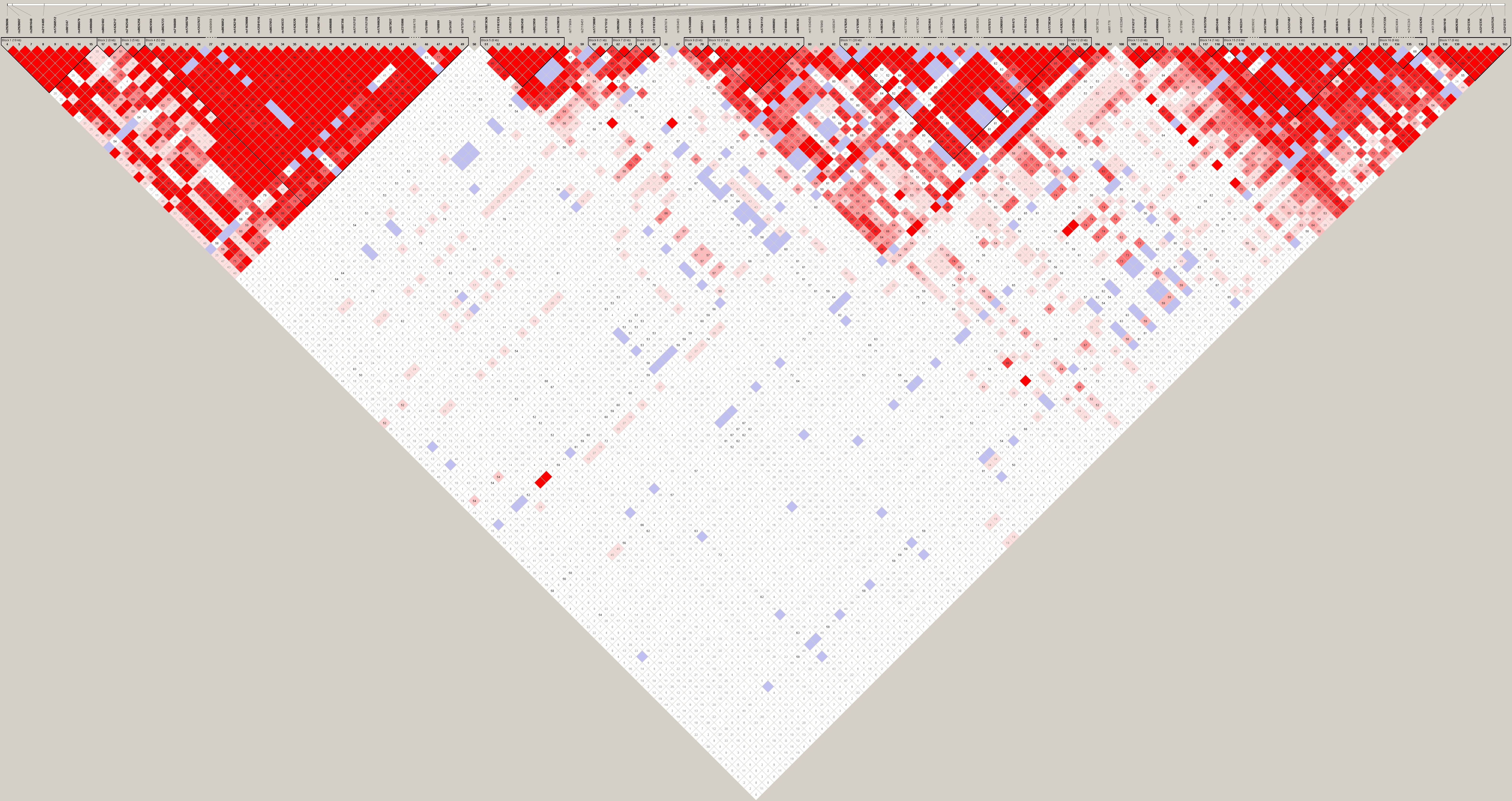
**

**Figure 2: LD structure of GABRB3 in the dataset investigated for EQ in the current study**

**
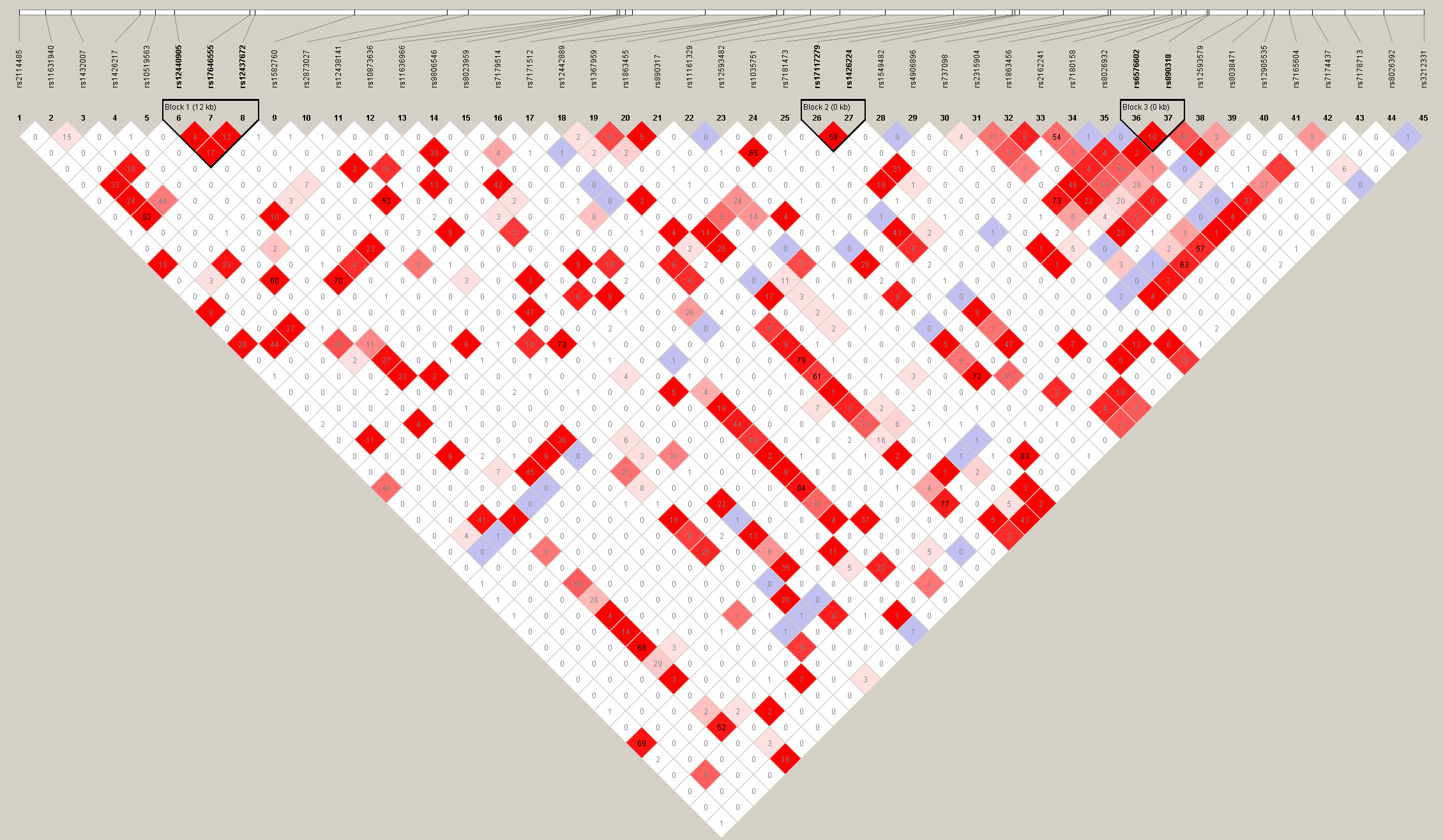
**

**Figure 3: LD structure of GABRB3 in the dataset investigated for EFT, MRT and RMET in the current study**

**
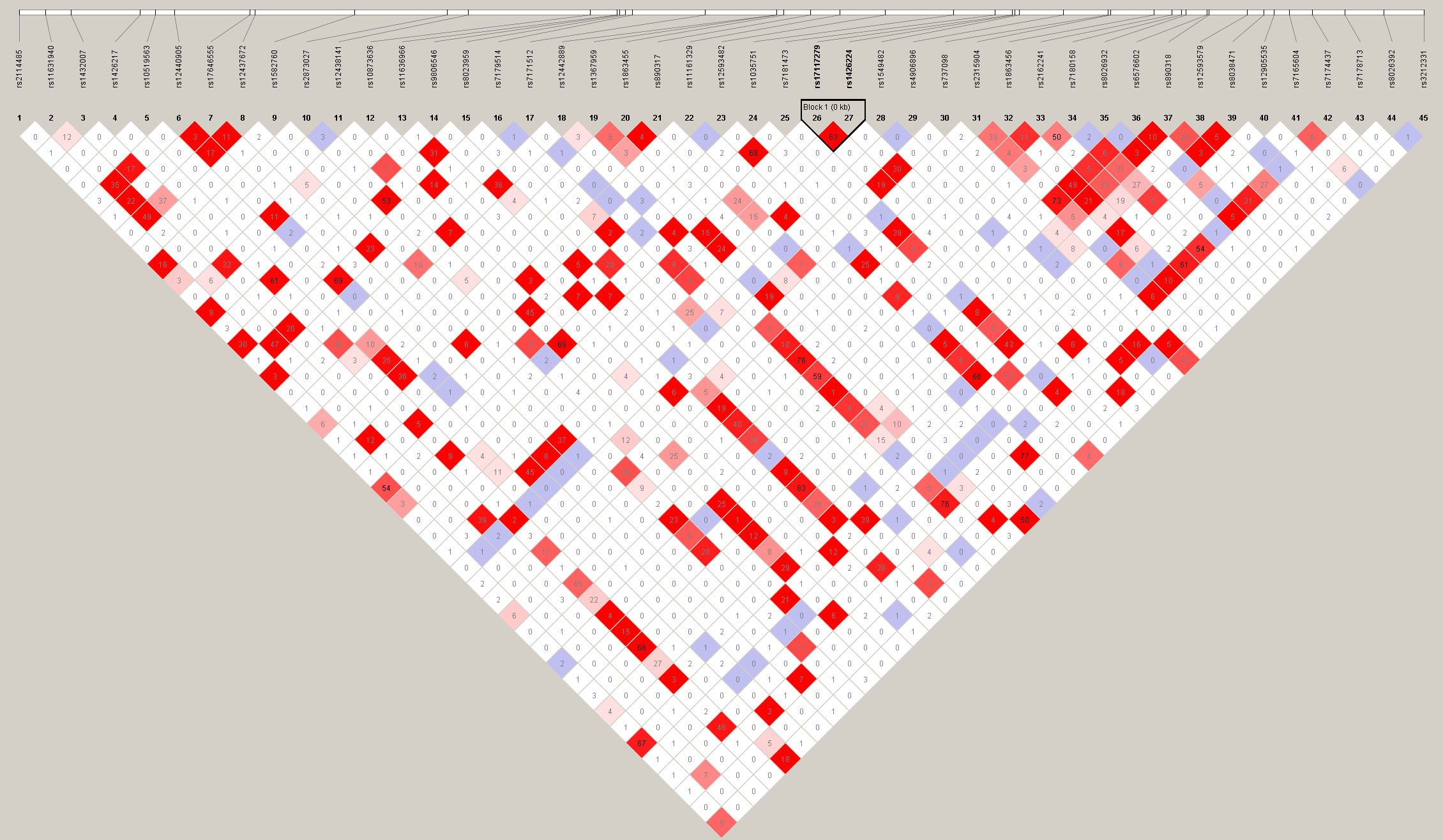
**
