# Supplementary material for: Genetic variation in GABRB3 is associated with Asperger syndrome and multiple endophenotypes relevant to autism
Source: Mol Autism. 2013 Dec 9;4:48. doi: 10.1186/2040-2392-4-48 (PMC3903107; doi:10.1186/2040-2392-4-48)
Supplement: Additional file 2 — GABRB 3 SNP-SNP interaction results for case–control (AS). AS, Asperger syndrome; SNP, Single nucleotide polymorphism. [file 2040-2392-4-48-S2.docx]

**Additional File 1**

**GABRB3 SNP-SNP interaction results for case-control (AS)**

| SNP1 | SNP2 | OR | P-value |
| --- | --- | --- | --- |
| rs2114485 | rs11631940 | 1.12E+00 | 7.72E-01 |
| rs2114485 | rs1432007 | 1.17E+00 | 5.95E-01 |
| rs2114485 | rs1426217 | 1.06E+00 | 8.37E-01 |
| rs2114485 | rs10519563 | 1.01E+00 | 9.79E-01 |
| rs2114485 | rs12440905 | 7.02E-01 | 7.10E-01 |
| rs2114485 | rs17646555 | 1.31E+00 | 6.26E-01 |
| rs2114485 | rs12437672 | 1.10E+00 | 7.30E-01 |
| rs2114485 | rs1582760 | 1.10E+00 | 8.45E-01 |
| rs2114485 | rs2873027 | 1.02E+00 | 9.42E-01 |
| rs2114485 | rs12438141 | 1.10E+00 | 7.80E-01 |
| rs2114485 | rs10873636 | 7.72E-01 | 3.91E-01 |
| rs2114485 | rs11636966 | 1.38E+00 | 2.31E-01 |
| rs2114485 | rs9806546 | 1.14E+00 | 7.27E-01 |
| rs2114485 | rs8023959 | 6.36E-01 | 2.79E-01 |
| rs2114485 | rs7179514 | 1.03E+00 | 9.05E-01 |
| rs2114485 | rs7171512 | 1.58E+00 | 9.66E-02 |
| rs2114485 | rs12442889 | 1.60E+00 | 1.05E-01 |
| rs2114485 | rs1367959 | 6.54E-01 | 1.32E-01 |
| rs2114485 | rs1863455 | 4.73E-01 | 8.74E-02 |
| rs2114485 | rs890317 | 1.63E+00 | 1.24E-01 |
| rs2114485 | rs11161329 | 9.82E-01 | 9.49E-01 |
| rs2114485 | rs12593482 | 9.61E-01 | 9.48E-01 |
| rs2114485 | rs1035751 | 1.26E+00 | 6.12E-01 |
| rs2114485 | rs7181473 | 1.16E+00 | 5.87E-01 |
| rs2114485 | rs17117279 | 1.97E+00 | 6.25E-02 |
| rs2114485 | rs1426224 | 7.83E-01 | 6.80E-01 |
| rs2114485 | rs1549482 | 7.80E-01 | 4.40E-01 |
| rs2114485 | rs4906896 | 1.39E+00 | 2.00E-01 |
| rs2114485 | rs737098 | 2.93E+00 | 5.14E-04 |
| rs2114485 | rs2315904 | 1.21E+00 | 6.31E-01 |
| rs2114485 | rs1863456 | 1.26E+00 | 3.75E-01 |
| rs2114485 | rs2162241 | 2.97E+00 | 1.23E-04 |
| rs2114485 | rs7180158 | 2.83E-01 | 4.59E-03 |
| rs2114485 | rs8026932 | 1.46E+00 | 5.71E-01 |
| rs2114485 | rs6576602 | 4.41E-01 | 1.33E-02 |
| rs2114485 | rs890318 | 1.26E+00 | 6.51E-01 |
| rs2114485 | rs12593579 | 4.22E-01 | 1.33E-02 |
| rs2114485 | rs8038471 | 7.43E-01 | 2.50E-01 |
| rs2114485 | rs12905535 | 2.70E+00 | 8.34E-04 |
| rs2114485 | rs7165604 | 2.19E-01 | 4.37E-03 |
| rs2114485 | rs7174437 | 2.45E-01 | 2.73E-02 |
| rs2114485 | rs7178713 | 2.47E+00 | 1.37E-03 |
| rs2114485 | rs8026392 | 1.24E+00 | 4.25E-01 |
| rs2114485 | rs3212331 | 8.23E-01 | 5.17E-01 |
| rs11631940 | rs1432007 | 9.50E-01 | 8.71E-01 |
| rs11631940 | rs1426217 | 1.11E+00 | 7.48E-01 |
| rs11631940 | rs10519563 | 8.42E-01 | 6.17E-01 |
| rs11631940 | rs12440905 | 7.99E+00 | 8.76E-02 |
| rs11631940 | rs17646555 | 6.54E-01 | 3.17E-01 |
| rs11631940 | rs12437672 | 1.38E+00 | 2.68E-01 |
| rs11631940 | rs1582760 | 8.17E-01 | 5.98E-01 |
| rs11631940 | rs2873027 | 6.95E-01 | 2.77E-01 |
| rs11631940 | rs12438141 | 9.30E-01 | 8.68E-01 |
| rs11631940 | rs10873636 | 1.19E+00 | 5.59E-01 |
| rs11631940 | rs11636966 | 8.14E-01 | 4.55E-01 |
| rs11631940 | rs9806546 | 1.44E+00 | 3.16E-01 |
| rs11631940 | rs8023959 | 8.72E-01 | 7.61E-01 |
| rs11631940 | rs7179514 | 9.42E-01 | 8.17E-01 |
| rs11631940 | rs7171512 | 7.75E-01 | 3.51E-01 |
| rs11631940 | rs12442889 | 6.25E-01 | 1.07E-01 |
| rs11631940 | rs1367959 | 8.34E-01 | 5.27E-01 |
| rs11631940 | rs1863455 | 2.35E+00 | 4.03E-02 |
| rs11631940 | rs890317 | 1.45E+00 | 1.75E-01 |
| rs11631940 | rs11161329 | 1.51E+00 | 9.85E-02 |
| rs11631940 | rs12593482 | 2.54E+00 | 1.65E-01 |
| rs11631940 | rs1035751 | 1.25E+00 | 6.34E-01 |
| rs11631940 | rs7181473 | 1.39E+00 | 2.27E-01 |
| rs11631940 | rs17117279 | 5.81E-01 | 1.47E-01 |
| rs11631940 | rs1426224 | 1.03E+00 | 9.66E-01 |
| rs11631940 | rs1549482 | 2.11E+00 | 1.52E-02 |
| rs11631940 | rs4906896 | 6.24E-01 | 7.64E-02 |
| rs11631940 | rs737098 | 5.44E-01 | 6.63E-02 |
| rs11631940 | rs2315904 | 5.06E-01 | 1.76E-01 |
| rs11631940 | rs1863456 | 7.62E-01 | 2.89E-01 |
| rs11631940 | rs2162241 | 5.12E-01 | 2.37E-02 |
| rs11631940 | rs7180158 | 1.30E+00 | 4.21E-01 |
| rs11631940 | rs8026932 | 1.96E+00 | 3.71E-01 |
| rs11631940 | rs6576602 | 1.07E+00 | 8.22E-01 |
| rs11631940 | rs890318 | 4.27E-01 | 1.50E-01 |
| rs11631940 | rs12593579 | 1.49E+00 | 1.95E-01 |
| rs11631940 | rs8038471 | 1.68E+00 | 5.98E-02 |
| rs11631940 | rs12905535 | 4.03E-01 | 7.30E-03 |
| rs11631940 | rs7165604 | 1.65E+00 | 2.50E-01 |
| rs11631940 | rs7174437 | 1.91E+00 | 1.68E-01 |
| rs11631940 | rs7178713 | 5.00E-01 | 2.71E-02 |
| rs11631940 | rs8026392 | 7.87E-01 | 3.87E-01 |
| rs11631940 | rs3212331 | 8.00E-01 | 4.34E-01 |
| rs1432007 | rs1426217 | 1.17E+00 | 5.11E-01 |
| rs1432007 | rs10519563 | 1.19E+00 | 6.03E-01 |
| rs1432007 | rs12440905 | 7.40E-10 | 9.99E-01 |
| rs1432007 | rs17646555 | 1.33E+00 | 4.47E-01 |
| rs1432007 | rs12437672 | 9.08E-01 | 7.17E-01 |
| rs1432007 | rs1582760 | 1.21E+00 | 5.95E-01 |
| rs1432007 | rs2873027 | 9.69E-01 | 8.91E-01 |
| rs1432007 | rs12438141 | 1.03E+00 | 9.36E-01 |
| rs1432007 | rs10873636 | 1.27E+00 | 3.64E-01 |
| rs1432007 | rs11636966 | 8.31E-01 | 4.24E-01 |
| rs1432007 | rs9806546 | 8.10E-01 | 5.40E-01 |
| rs1432007 | rs8023959 | 1.83E+00 | 1.11E-01 |
| rs1432007 | rs7179514 | 9.54E-01 | 8.35E-01 |
| rs1432007 | rs7171512 | 7.96E-01 | 3.29E-01 |
| rs1432007 | rs12442889 | 9.42E-01 | 8.13E-01 |
| rs1432007 | rs1367959 | 1.66E+00 | 4.88E-02 |
| rs1432007 | rs1863455 | 1.03E+00 | 9.28E-01 |
| rs1432007 | rs890317 | 4.77E-01 | 7.53E-03 |
| rs1432007 | rs11161329 | 7.24E-01 | 1.81E-01 |
| rs1432007 | rs12593482 | 6.52E-01 | 4.96E-01 |
| rs1432007 | rs1035751 | 7.06E-01 | 4.25E-01 |
| rs1432007 | rs7181473 | 7.57E-01 | 2.49E-01 |
| rs1432007 | rs17117279 | 9.15E-01 | 7.79E-01 |
| rs1432007 | rs1426224 | 1.65E+00 | 3.86E-01 |
| rs1432007 | rs1549482 | 7.01E-01 | 2.10E-01 |
| rs1432007 | rs4906896 | 1.06E+00 | 8.00E-01 |
| rs1432007 | rs737098 | 6.49E-01 | 1.05E-01 |
| rs1432007 | rs2315904 | 1.14E+00 | 7.19E-01 |
| rs1432007 | rs1863456 | 9.27E-01 | 7.27E-01 |
| rs1432007 | rs2162241 | 6.54E-01 | 7.51E-02 |
| rs1432007 | rs7180158 | 1.77E+00 | 8.40E-02 |
| rs1432007 | rs8026932 | 3.92E-01 | 1.55E-01 |
| rs1432007 | rs6576602 | 1.52E+00 | 1.25E-01 |
| rs1432007 | rs890318 | 1.23E+00 | 6.37E-01 |
| rs1432007 | rs12593579 | 1.29E+00 | 3.84E-01 |
| rs1432007 | rs8038471 | 9.29E-01 | 7.40E-01 |
| rs1432007 | rs12905535 | 8.44E-01 | 5.24E-01 |
| rs1432007 | rs7165604 | 1.85E+00 | 1.05E-01 |
| rs1432007 | rs7174437 | 1.62E+00 | 2.59E-01 |
| rs1432007 | rs7178713 | 7.51E-01 | 2.62E-01 |
| rs1432007 | rs8026392 | 9.82E-01 | 9.41E-01 |
| rs1432007 | rs3212331 | 1.24E+00 | 3.92E-01 |
| rs1426217 | rs10519563 | 1.01E+00 | 9.79E-01 |
| rs1426217 | rs12440905 | 2.43E+00 | 4.27E-01 |
| rs1426217 | rs17646555 | 1.50E+00 | 4.41E-01 |
| rs1426217 | rs12437672 | 1.15E+00 | 5.76E-01 |
| rs1426217 | rs1582760 | 1.12E+00 | 7.68E-01 |
| rs1426217 | rs2873027 | 9.60E-01 | 8.66E-01 |
| rs1426217 | rs12438141 | 8.21E-01 | 6.04E-01 |
| rs1426217 | rs10873636 | 9.44E-01 | 8.15E-01 |
| rs1426217 | rs11636966 | 9.29E-01 | 7.43E-01 |
| rs1426217 | rs9806546 | 1.13E+00 | 7.01E-01 |
| rs1426217 | rs8023959 | 7.19E-01 | 3.43E-01 |
| rs1426217 | rs7179514 | 1.08E+00 | 7.38E-01 |
| rs1426217 | rs7171512 | 7.51E-01 | 2.12E-01 |
| rs1426217 | rs12442889 | 8.22E-01 | 3.94E-01 |
| rs1426217 | rs1367959 | 7.16E-01 | 1.37E-01 |
| rs1426217 | rs1863455 | 2.03E+00 | 5.33E-02 |
| rs1426217 | rs890317 | 1.28E+00 | 3.31E-01 |
| rs1426217 | rs11161329 | 1.62E+00 | 3.40E-02 |
| rs1426217 | rs12593482 | 1.76E+00 | 3.64E-01 |
| rs1426217 | rs1035751 | 1.25E+00 | 5.68E-01 |
| rs1426217 | rs7181473 | 1.18E+00 | 4.82E-01 |
| rs1426217 | rs17117279 | 6.18E-01 | 1.20E-01 |
| rs1426217 | rs1426224 | 1.69E+00 | 2.68E-01 |
| rs1426217 | rs1549482 | 1.83E+00 | 2.28E-02 |
| rs1426217 | rs4906896 | 9.12E-01 | 6.68E-01 |
| rs1426217 | rs737098 | 1.17E+00 | 5.33E-01 |
| rs1426217 | rs2315904 | 6.40E-01 | 2.03E-01 |
| rs1426217 | rs1863456 | 9.84E-01 | 9.40E-01 |
| rs1426217 | rs2162241 | 1.07E+00 | 7.77E-01 |
| rs1426217 | rs7180158 | 7.61E-01 | 3.77E-01 |
| rs1426217 | rs8026932 | 1.12E+00 | 8.61E-01 |
| rs1426217 | rs6576602 | 7.79E-01 | 3.12E-01 |
| rs1426217 | rs890318 | 7.77E-01 | 5.04E-01 |
| rs1426217 | rs12593579 | 8.82E-01 | 6.60E-01 |
| rs1426217 | rs8038471 | 1.10E+00 | 6.68E-01 |
| rs1426217 | rs12905535 | 1.01E+00 | 9.57E-01 |
| rs1426217 | rs7165604 | 7.45E-01 | 4.15E-01 |
| rs1426217 | rs7174437 | 8.85E-01 | 7.49E-01 |
| rs1426217 | rs7178713 | 1.19E+00 | 4.68E-01 |
| rs1426217 | rs8026392 | 1.00E+00 | 9.98E-01 |
| rs1426217 | rs3212331 | 7.87E-01 | 3.19E-01 |
| rs10519563 | rs12440905 | 1.44E+01 | 5.76E-02 |
| rs10519563 | rs17646555 | 2.53E+00 | 1.41E-01 |
| rs10519563 | rs12437672 | 8.91E-01 | 7.43E-01 |
| rs10519563 | rs1582760 | 3.77E+00 | 2.40E-02 |
| rs10519563 | rs2873027 | 6.93E-01 | 2.99E-01 |
| rs10519563 | rs12438141 | 1.07E+00 | 8.91E-01 |
| rs10519563 | rs10873636 | 1.29E+00 | 4.15E-01 |
| rs10519563 | rs11636966 | 8.29E-01 | 5.04E-01 |
| rs10519563 | rs9806546 | 1.56E+00 | 2.21E-01 |
| rs10519563 | rs8023959 | 7.62E-01 | 6.11E-01 |
| rs10519563 | rs7179514 | 1.01E+00 | 9.60E-01 |
| rs10519563 | rs7171512 | 6.83E-01 | 1.80E-01 |
| rs10519563 | rs12442889 | 7.13E-01 | 2.77E-01 |
| rs10519563 | rs1367959 | 9.49E-01 | 8.65E-01 |
| rs10519563 | rs1863455 | 1.60E+00 | 2.63E-01 |
| rs10519563 | rs890317 | 1.30E+00 | 3.71E-01 |
| rs10519563 | rs11161329 | 1.27E+00 | 3.59E-01 |
| rs10519563 | rs12593482 | 2.08E+00 | 2.86E-01 |
| rs10519563 | rs1035751 | 1.44E+00 | 4.66E-01 |
| rs10519563 | rs7181473 | 1.24E+00 | 4.41E-01 |
| rs10519563 | rs17117279 | 6.90E-01 | 3.23E-01 |
| rs10519563 | rs1426224 | 3.65E-01 | 2.49E-01 |
| rs10519563 | rs1549482 | 1.77E+00 | 6.84E-02 |
| rs10519563 | rs4906896 | 5.48E-01 | 3.37E-02 |
| rs10519563 | rs737098 | 5.35E-01 | 7.83E-02 |
| rs10519563 | rs2315904 | 3.73E-01 | 6.70E-02 |
| rs10519563 | rs1863456 | 6.43E-01 | 9.85E-02 |
| rs10519563 | rs2162241 | 4.37E-01 | 9.85E-03 |
| rs10519563 | rs7180158 | 1.03E+00 | 9.33E-01 |
| rs10519563 | rs8026932 | 1.71E+00 | 5.61E-01 |
| rs10519563 | rs6576602 | 8.06E-01 | 4.71E-01 |
| rs10519563 | rs890318 | 2.90E-01 | 6.05E-02 |
| rs10519563 | rs12593579 | 1.17E+00 | 6.13E-01 |
| rs10519563 | rs8038471 | 1.96E+00 | 1.89E-02 |
| rs10519563 | rs12905535 | 4.47E-01 | 2.24E-02 |
| rs10519563 | rs7165604 | 1.22E+00 | 6.39E-01 |
| rs10519563 | rs7174437 | 1.45E+00 | 4.29E-01 |
| rs10519563 | rs7178713 | 5.29E-01 | 5.26E-02 |
| rs10519563 | rs8026392 | 8.04E-01 | 4.50E-01 |
| rs10519563 | rs3212331 | 6.56E-01 | 1.70E-01 |
| rs12440905 | rs17646555 | 1.11E-09 | 9.99E-01 |
| rs12440905 | rs12437672 | 3.93E+00 | 2.88E-01 |
| rs12440905 | rs1582760 | 9.66E-01 | 9.82E-01 |
| rs12440905 | rs2873027 | 5.94E-10 | 9.98E-01 |
| rs12440905 | rs12438141 | 9.68E-10 | 9.99E-01 |
| rs12440905 | rs10873636 | 4.58E+00 | 2.28E-01 |
| rs12440905 | rs11636966 | 5.57E-01 | 6.10E-01 |
| rs12440905 | rs9806546 | 1.78E+00 | 6.07E-01 |
| rs12440905 | rs8023959 | 2.69E+00 | 3.83E-01 |
| rs12440905 | rs7179514 | 1.40E-01 | 1.41E-01 |
| rs12440905 | rs7171512 | 1.30E+00 | 8.11E-01 |
| rs12440905 | rs12442889 | 6.49E-01 | 6.68E-01 |
| rs12440905 | rs1367959 | 6.70E-01 | 7.18E-01 |
| rs12440905 | rs1863455 | 1.75E+00 | 5.42E-01 |
| rs12440905 | rs890317 | 1.12E+00 | 9.05E-01 |
| rs12440905 | rs11161329 | 1.53E+00 | 6.13E-01 |
| rs12440905 | rs12593482 | 1.44E+00 | 7.12E-01 |
| rs12440905 | rs1035751 | 8.44E-01 | 8.99E-01 |
| rs12440905 | rs7181473 | 1.62E+00 | 6.15E-01 |
| rs12440905 | rs17117279 | 7.60E-01 | 8.36E-01 |
| rs12440905 | rs1426224 | 1.21E-09 | 9.99E-01 |
| rs12440905 | rs1549482 | 2.50E+00 | 4.16E-01 |
| rs12440905 | rs4906896 | 7.67E-01 | 7.59E-01 |
| rs12440905 | rs737098 | 6.57E-01 | 6.41E-01 |
| rs12440905 | rs2315904 | 5.35E+00 | 1.51E-01 |
| rs12440905 | rs1863456 | 7.04E-01 | 6.61E-01 |
| rs12440905 | rs2162241 | 7.30E-01 | 7.27E-01 |
| rs12440905 | rs7180158 | 5.99E-10 | 9.99E-01 |
| rs12440905 | rs8026932 | 9.06E-01 | 9.45E-01 |
| rs12440905 | rs6576602 | 9.26E-01 | 9.20E-01 |
| rs12440905 | rs890318 | 1.60E+00 | 6.17E-01 |
| rs12440905 | rs12593579 | 9.83E-01 | 9.88E-01 |
| rs12440905 | rs8038471 | 1.66E+00 | 4.98E-01 |
| rs12440905 | rs12905535 | 3.68E-01 | 3.83E-01 |
| rs12440905 | rs7165604 | 5.24E-10 | 9.99E-01 |
| rs12440905 | rs7174437 | 6.31E-10 | 9.99E-01 |
| rs12440905 | rs7178713 | 8.92E-01 | 8.96E-01 |
| rs12440905 | rs8026392 | 5.21E-01 | 4.69E-01 |
| rs12440905 | rs3212331 | 1.92E+00 | 3.70E-01 |
| rs17646555 | rs12437672 | 3.05E+00 | 7.77E-02 |
| rs17646555 | rs1582760 | 9.18E-01 | 8.96E-01 |
| rs17646555 | rs2873027 | 7.54E-01 | 5.99E-01 |
| rs17646555 | rs12438141 | 3.28E-01 | 1.78E-01 |
| rs17646555 | rs10873636 | 3.95E-01 | 5.76E-02 |
| rs17646555 | rs11636966 | 1.24E+00 | 6.42E-01 |
| rs17646555 | rs9806546 | 3.39E-01 | 1.08E-01 |
| rs17646555 | rs8023959 | 6.45E-01 | 4.96E-01 |
| rs17646555 | rs7179514 | 1.13E+00 | 7.67E-01 |
| rs17646555 | rs7171512 | 1.57E+00 | 2.83E-01 |
| rs17646555 | rs12442889 | 5.08E-01 | 1.09E-01 |
| rs17646555 | rs1367959 | 9.62E-01 | 9.18E-01 |
| rs17646555 | rs1863455 | 1.09E+01 | 2.51E-04 |
| rs17646555 | rs890317 | 7.62E-01 | 5.35E-01 |
| rs17646555 | rs11161329 | 1.85E+00 | 9.74E-02 |
| rs17646555 | rs12593482 | 9.56E-01 | 9.71E-01 |
| rs17646555 | rs1035751 | 2.27E-01 | 1.64E-01 |
| rs17646555 | rs7181473 | 1.32E+00 | 5.19E-01 |
| rs17646555 | rs17117279 | 2.58E-01 | 9.24E-02 |
| rs17646555 | rs1426224 | 5.77E+00 | 4.42E-02 |
| rs17646555 | rs1549482 | 2.10E+00 | 1.15E-01 |
| rs17646555 | rs4906896 | 8.74E-01 | 7.28E-01 |
| rs17646555 | rs737098 | 1.04E+00 | 9.31E-01 |
| rs17646555 | rs2315904 | 9.52E-01 | 9.27E-01 |
| rs17646555 | rs1863456 | 8.74E-01 | 7.31E-01 |
| rs17646555 | rs2162241 | 9.97E-01 | 9.93E-01 |
| rs17646555 | rs7180158 | 8.52E-01 | 7.74E-01 |
| rs17646555 | rs8026932 | 4.95E-01 | 5.15E-01 |
| rs17646555 | rs6576602 | 8.22E-01 | 6.74E-01 |
| rs17646555 | rs890318 | 9.25E-01 | 9.13E-01 |
| rs17646555 | rs12593579 | 7.73E-01 | 6.19E-01 |
| rs17646555 | rs8038471 | 1.10E+00 | 8.09E-01 |
| rs17646555 | rs12905535 | 1.17E+00 | 7.14E-01 |
| rs17646555 | rs7165604 | 4.06E-01 | 4.56E-01 |
| rs17646555 | rs7174437 | 3.11E-01 | 3.14E-01 |
| rs17646555 | rs7178713 | 1.02E+00 | 9.73E-01 |
| rs17646555 | rs8026392 | 7.09E-01 | 4.21E-01 |
| rs17646555 | rs3212331 | 9.93E-01 | 9.86E-01 |
| rs12437672 | rs1582760 | 3.54E+00 | 4.32E-02 |
| rs12437672 | rs2873027 | 7.55E-01 | 3.16E-01 |
| rs12437672 | rs12438141 | 9.03E-01 | 8.21E-01 |
| rs12437672 | rs10873636 | 1.22E+00 | 4.82E-01 |
| rs12437672 | rs11636966 | 8.89E-01 | 6.37E-01 |
| rs12437672 | rs9806546 | 1.81E+00 | 7.69E-02 |
| rs12437672 | rs8023959 | 6.68E-01 | 3.73E-01 |
| rs12437672 | rs7179514 | 9.22E-01 | 7.34E-01 |
| rs12437672 | rs7171512 | 9.16E-01 | 7.25E-01 |
| rs12437672 | rs12442889 | 9.65E-01 | 8.97E-01 |
| rs12437672 | rs1367959 | 8.57E-01 | 5.74E-01 |
| rs12437672 | rs1863455 | 1.02E+00 | 9.55E-01 |
| rs12437672 | rs890317 | 1.47E+00 | 1.76E-01 |
| rs12437672 | rs11161329 | 1.20E+00 | 4.85E-01 |
| rs12437672 | rs12593482 | 1.24E+00 | 7.34E-01 |
| rs12437672 | rs1035751 | 1.77E+00 | 2.29E-01 |
| rs12437672 | rs7181473 | 1.40E+00 | 1.92E-01 |
| rs12437672 | rs17117279 | 8.58E-01 | 6.53E-01 |
| rs12437672 | rs1426224 | 6.04E-01 | 4.52E-01 |
| rs12437672 | rs1549482 | 1.93E+00 | 3.46E-02 |
| rs12437672 | rs4906896 | 8.16E-01 | 4.15E-01 |
| rs12437672 | rs737098 | 1.12E+00 | 6.95E-01 |
| rs12437672 | rs2315904 | 5.97E-01 | 2.42E-01 |
| rs12437672 | rs1863456 | 8.34E-01 | 4.39E-01 |
| rs12437672 | rs2162241 | 9.20E-01 | 7.46E-01 |
| rs12437672 | rs7180158 | 6.64E-01 | 2.03E-01 |
| rs12437672 | rs8026932 | 4.05E+00 | 2.50E-01 |
| rs12437672 | rs6576602 | 5.87E-01 | 6.37E-02 |
| rs12437672 | rs890318 | 3.31E-01 | 4.91E-02 |
| rs12437672 | rs12593579 | 8.61E-01 | 6.22E-01 |
| rs12437672 | rs8038471 | 1.23E+00 | 4.00E-01 |
| rs12437672 | rs12905535 | 1.09E+00 | 7.62E-01 |
| rs12437672 | rs7165604 | 6.15E-01 | 2.04E-01 |
| rs12437672 | rs7174437 | 7.92E-01 | 5.98E-01 |
| rs12437672 | rs7178713 | 1.15E+00 | 6.01E-01 |
| rs12437672 | rs8026392 | 1.13E+00 | 6.35E-01 |
| rs12437672 | rs3212331 | 5.67E-01 | 4.91E-02 |
| rs1582760 | rs2873027 | 4.97E-01 | 1.72E-01 |
| rs1582760 | rs12438141 | 2.94E-01 | 1.34E-01 |
| rs1582760 | rs10873636 | 6.47E-01 | 2.84E-01 |
| rs1582760 | rs11636966 | 1.08E+00 | 8.61E-01 |
| rs1582760 | rs9806546 | 5.06E-01 | 2.20E-01 |
| rs1582760 | rs8023959 | 1.03E+00 | 9.63E-01 |
| rs1582760 | rs7179514 | 9.74E-01 | 9.49E-01 |
| rs1582760 | rs7171512 | 1.60E+00 | 2.45E-01 |
| rs1582760 | rs12442889 | 5.33E-01 | 1.05E-01 |
| rs1582760 | rs1367959 | 9.42E-01 | 8.67E-01 |
| rs1582760 | rs1863455 | 8.56E+00 | 2.38E-04 |
| rs1582760 | rs890317 | 7.44E-01 | 4.74E-01 |
| rs1582760 | rs11161329 | 1.82E+00 | 8.95E-02 |
| rs1582760 | rs12593482 | 1.49E+00 | 6.15E-01 |
| rs1582760 | rs1035751 | 3.38E-01 | 1.66E-01 |
| rs1582760 | rs7181473 | 1.19E+00 | 6.74E-01 |
| rs1582760 | rs17117279 | 3.43E-01 | 1.17E-01 |
| rs1582760 | rs1426224 | 4.81E+00 | 6.40E-02 |
| rs1582760 | rs1549482 | 1.79E+00 | 2.05E-01 |
| rs1582760 | rs4906896 | 8.42E-01 | 6.32E-01 |
| rs1582760 | rs737098 | 9.92E-01 | 9.83E-01 |
| rs1582760 | rs2315904 | 1.34E+00 | 5.24E-01 |
| rs1582760 | rs1863456 | 8.41E-01 | 6.28E-01 |
| rs1582760 | rs2162241 | 1.08E+00 | 8.31E-01 |
| rs1582760 | rs7180158 | 4.98E-01 | 1.99E-01 |
| rs1582760 | rs8026932 | 6.76E-01 | 6.53E-01 |
| rs1582760 | rs6576602 | 7.37E-01 | 4.59E-01 |
| rs1582760 | rs890318 | 1.26E+00 | 6.99E-01 |
| rs1582760 | rs12593579 | 6.42E-01 | 3.51E-01 |
| rs1582760 | rs8038471 | 1.23E+00 | 5.68E-01 |
| rs1582760 | rs12905535 | 9.93E-01 | 9.86E-01 |
| rs1582760 | rs7165604 | 2.00E-01 | 1.22E-01 |
| rs1582760 | rs7174437 | 1.89E-01 | 1.18E-01 |
| rs1582760 | rs7178713 | 1.05E+00 | 9.12E-01 |
| rs1582760 | rs8026392 | 6.68E-01 | 3.11E-01 |
| rs1582760 | rs3212331 | 1.29E+00 | 4.67E-01 |
| rs2873027 | rs12438141 | 1.90E+00 | 1.07E-01 |
| rs2873027 | rs10873636 | 9.13E-01 | 7.16E-01 |
| rs2873027 | rs11636966 | 1.03E+00 | 9.06E-01 |
| rs2873027 | rs9806546 | 5.50E-01 | 8.82E-02 |
| rs2873027 | rs8023959 | 1.42E+00 | 3.18E-01 |
| rs2873027 | rs7179514 | 1.06E+00 | 7.76E-01 |
| rs2873027 | rs7171512 | 1.09E+00 | 7.00E-01 |
| rs2873027 | rs12442889 | 1.14E+00 | 5.59E-01 |
| rs2873027 | rs1367959 | 1.40E+00 | 1.42E-01 |
| rs2873027 | rs1863455 | 6.26E-01 | 1.94E-01 |
| rs2873027 | rs890317 | 6.81E-01 | 1.40E-01 |
| rs2873027 | rs11161329 | 6.60E-01 | 6.46E-02 |
| rs2873027 | rs12593482 | 6.57E-01 | 4.85E-01 |
| rs2873027 | rs1035751 | 5.97E-01 | 2.50E-01 |
| rs2873027 | rs7181473 | 7.55E-01 | 2.27E-01 |
| rs2873027 | rs17117279 | 1.53E+00 | 1.56E-01 |
| rs2873027 | rs1426224 | 5.56E-01 | 2.79E-01 |
| rs2873027 | rs1549482 | 4.09E-01 | 2.72E-03 |
| rs2873027 | rs4906896 | 9.75E-01 | 9.06E-01 |
| rs2873027 | rs737098 | 6.68E-01 | 1.06E-01 |
| rs2873027 | rs2315904 | 1.04E+00 | 9.19E-01 |
| rs2873027 | rs1863456 | 9.63E-01 | 8.60E-01 |
| rs2873027 | rs2162241 | 6.68E-01 | 8.50E-02 |
| rs2873027 | rs7180158 | 2.14E+00 | 1.70E-02 |
| rs2873027 | rs8026932 | 4.69E-01 | 3.48E-01 |
| rs2873027 | rs6576602 | 1.68E+00 | 4.57E-02 |
| rs2873027 | rs890318 | 1.18E+00 | 6.98E-01 |
| rs2873027 | rs12593579 | 1.52E+00 | 1.66E-01 |
| rs2873027 | rs8038471 | 1.01E+00 | 9.66E-01 |
| rs2873027 | rs12905535 | 7.42E-01 | 2.36E-01 |
| rs2873027 | rs7165604 | 2.14E+00 | 3.01E-02 |
| rs2873027 | rs7174437 | 1.80E+00 | 1.35E-01 |
| rs2873027 | rs7178713 | 6.12E-01 | 4.77E-02 |
| rs2873027 | rs8026392 | 9.41E-01 | 7.92E-01 |
| rs2873027 | rs3212331 | 1.18E+00 | 5.16E-01 |
| rs12438141 | rs10873636 | 1.15E+00 | 6.72E-01 |
| rs12438141 | rs11636966 | 1.12E+00 | 7.18E-01 |
| rs12438141 | rs9806546 | 1.20E+00 | 6.89E-01 |
| rs12438141 | rs8023959 | 9.86E-01 | 9.75E-01 |
| rs12438141 | rs7179514 | 1.11E+00 | 7.35E-01 |
| rs12438141 | rs7171512 | 7.97E-01 | 4.94E-01 |
| rs12438141 | rs12442889 | 1.73E+00 | 1.06E-01 |
| rs12438141 | rs1367959 | 6.17E-01 | 1.24E-01 |
| rs12438141 | rs1863455 | 2.83E-01 | 5.59E-02 |
| rs12438141 | rs890317 | 1.70E+00 | 1.50E-01 |
| rs12438141 | rs11161329 | 1.08E+00 | 8.19E-01 |
| rs12438141 | rs12593482 | 1.36E+00 | 6.61E-01 |
| rs12438141 | rs1035751 | 2.05E+00 | 1.66E-01 |
| rs12438141 | rs7181473 | 8.86E-01 | 7.19E-01 |
| rs12438141 | rs17117279 | 1.38E+00 | 4.50E-01 |
| rs12438141 | rs1426224 | 2.18E+00 | 2.16E-01 |
| rs12438141 | rs1549482 | 8.45E-01 | 6.55E-01 |
| rs12438141 | rs4906896 | 1.75E+00 | 7.03E-02 |
| rs12438141 | rs737098 | 2.41E+00 | 1.39E-02 |
| rs12438141 | rs2315904 | 1.01E+00 | 9.90E-01 |
| rs12438141 | rs1863456 | 1.80E+00 | 6.38E-02 |
| rs12438141 | rs2162241 | 2.95E+00 | 1.14E-03 |
| rs12438141 | rs7180158 | 4.95E-01 | 1.71E-01 |
| rs12438141 | rs8026932 | 3.52E+00 | 1.53E-01 |
| rs12438141 | rs6576602 | 7.75E-01 | 4.98E-01 |
| rs12438141 | rs890318 | 1.73E+00 | 2.96E-01 |
| rs12438141 | rs12593579 | 8.34E-01 | 6.55E-01 |
| rs12438141 | rs8038471 | 5.03E-01 | 2.97E-02 |
| rs12438141 | rs12905535 | 2.29E+00 | 2.01E-02 |
| rs12438141 | rs7165604 | 6.13E-01 | 3.98E-01 |
| rs12438141 | rs7174437 | 7.40E-01 | 6.15E-01 |
| rs12438141 | rs7178713 | 3.77E+00 | 2.21E-04 |
| rs12438141 | rs8026392 | 1.56E+00 | 1.72E-01 |
| rs12438141 | rs3212331 | 1.21E+00 | 5.54E-01 |
| rs10873636 | rs11636966 | 6.20E-01 | 1.27E-01 |
| rs10873636 | rs9806546 | 1.49E+00 | 3.37E-01 |
| rs10873636 | rs8023959 | 1.36E+00 | 4.91E-01 |
| rs10873636 | rs7179514 | 6.98E-01 | 2.23E-01 |
| rs10873636 | rs7171512 | 6.89E-01 | 2.33E-01 |
| rs10873636 | rs12442889 | 9.23E-01 | 7.83E-01 |
| rs10873636 | rs1367959 | 1.04E+00 | 8.86E-01 |
| rs10873636 | rs1863455 | 9.36E-01 | 8.82E-01 |
| rs10873636 | rs890317 | 1.05E+00 | 8.77E-01 |
| rs10873636 | rs11161329 | 1.10E+00 | 7.17E-01 |
| rs10873636 | rs12593482 | 2.04E+00 | 2.48E-01 |
| rs10873636 | rs1035751 | 8.65E-01 | 7.66E-01 |
| rs10873636 | rs7181473 | 6.86E-01 | 1.65E-01 |
| rs10873636 | rs17117279 | 3.84E-01 | 2.31E-02 |
| rs10873636 | rs1426224 | 1.91E+00 | 2.04E-01 |
| rs10873636 | rs1549482 | 1.03E+00 | 9.19E-01 |
| rs10873636 | rs4906896 | 1.15E+00 | 5.84E-01 |
| rs10873636 | rs737098 | 1.17E+00 | 5.77E-01 |
| rs10873636 | rs2315904 | 8.18E-01 | 6.36E-01 |
| rs10873636 | rs1863456 | 1.37E+00 | 2.02E-01 |
| rs10873636 | rs2162241 | 1.23E+00 | 4.10E-01 |
| rs10873636 | rs7180158 | 1.32E+00 | 4.21E-01 |
| rs10873636 | rs8026932 | 4.81E+00 | 5.00E-02 |
| rs10873636 | rs6576602 | 1.01E+00 | 9.80E-01 |
| rs10873636 | rs890318 | 7.54E-01 | 5.57E-01 |
| rs10873636 | rs12593579 | 1.87E+00 | 5.62E-02 |
| rs10873636 | rs8038471 | 8.50E-01 | 5.02E-01 |
| rs10873636 | rs12905535 | 9.31E-01 | 7.99E-01 |
| rs10873636 | rs7165604 | 1.47E+00 | 3.47E-01 |
| rs10873636 | rs7174437 | 1.30E+00 | 5.40E-01 |
| rs10873636 | rs7178713 | 1.18E+00 | 5.25E-01 |
| rs10873636 | rs8026392 | 1.25E+00 | 3.60E-01 |
| rs10873636 | rs3212331 | 9.17E-01 | 7.55E-01 |
| rs11636966 | rs9806546 | 6.64E-01 | 3.36E-01 |
| rs11636966 | rs8023959 | 6.51E-01 | 3.67E-01 |
| rs11636966 | rs7179514 | 1.14E+00 | 5.62E-01 |
| rs11636966 | rs7171512 | 9.41E-01 | 7.86E-01 |
| rs11636966 | rs12442889 | 1.34E+00 | 2.97E-01 |
| rs11636966 | rs1367959 | 7.62E-01 | 2.58E-01 |
| rs11636966 | rs1863455 | 1.06E+00 | 8.72E-01 |
| rs11636966 | rs890317 | 8.48E-01 | 5.37E-01 |
| rs11636966 | rs11161329 | 8.04E-01 | 3.67E-01 |
| rs11636966 | rs12593482 | 8.80E-01 | 8.31E-01 |
| rs11636966 | rs1035751 | 1.96E+00 | 1.18E-01 |
| rs11636966 | rs7181473 | 1.29E+00 | 3.18E-01 |
| rs11636966 | rs17117279 | 1.17E+00 | 6.19E-01 |
| rs11636966 | rs1426224 | 9.21E-01 | 8.88E-01 |
| rs11636966 | rs1549482 | 1.38E+00 | 2.85E-01 |
| rs11636966 | rs4906896 | 9.57E-01 | 8.44E-01 |
| rs11636966 | rs737098 | 8.15E-01 | 4.60E-01 |
| rs11636966 | rs2315904 | 1.50E+00 | 2.78E-01 |
| rs11636966 | rs1863456 | 8.89E-01 | 6.07E-01 |
| rs11636966 | rs2162241 | 9.75E-01 | 9.17E-01 |
| rs11636966 | rs7180158 | 1.05E+00 | 8.68E-01 |
| rs11636966 | rs8026932 | 5.22E-01 | 4.03E-01 |
| rs11636966 | rs6576602 | 1.12E+00 | 6.57E-01 |
| rs11636966 | rs890318 | 1.46E+00 | 3.51E-01 |
| rs11636966 | rs12593579 | 8.58E-01 | 6.03E-01 |
| rs11636966 | rs8038471 | 1.16E+00 | 5.11E-01 |
| rs11636966 | rs12905535 | 9.33E-01 | 7.99E-01 |
| rs11636966 | rs7165604 | 8.41E-01 | 6.49E-01 |
| rs11636966 | rs7174437 | 6.87E-01 | 3.65E-01 |
| rs11636966 | rs7178713 | 8.67E-01 | 5.82E-01 |
| rs11636966 | rs8026392 | 6.48E-01 | 7.27E-02 |
| rs11636966 | rs3212331 | 1.24E+00 | 4.08E-01 |
| rs9806546 | rs8023959 | 1.65E+00 | 3.85E-01 |
| rs9806546 | rs7179514 | 6.87E-01 | 3.53E-01 |
| rs9806546 | rs7171512 | 7.45E-01 | 4.78E-01 |
| rs9806546 | rs12442889 | 1.09E+00 | 8.22E-01 |
| rs9806546 | rs1367959 | 1.26E+00 | 5.35E-01 |
| rs9806546 | rs1863455 | 6.66E-01 | 4.89E-01 |
| rs9806546 | rs890317 | 1.00E+00 | 9.96E-01 |
| rs9806546 | rs11161329 | 9.42E-01 | 8.61E-01 |
| rs9806546 | rs12593482 | 2.28E+00 | 2.53E-01 |
| rs9806546 | rs1035751 | 8.55E-01 | 7.74E-01 |
| rs9806546 | rs7181473 | 6.70E-01 | 2.45E-01 |
| rs9806546 | rs17117279 | 2.05E-01 | 1.64E-02 |
| rs9806546 | rs1426224 | 2.17E+00 | 2.63E-01 |
| rs9806546 | rs1549482 | 1.19E+00 | 6.33E-01 |
| rs9806546 | rs4906896 | 7.29E-01 | 3.41E-01 |
| rs9806546 | rs737098 | 9.98E-01 | 9.95E-01 |
| rs9806546 | rs2315904 | 8.42E-01 | 7.40E-01 |
| rs9806546 | rs1863456 | 8.09E-01 | 5.05E-01 |
| rs9806546 | rs2162241 | 9.58E-01 | 8.99E-01 |
| rs9806546 | rs7180158 | 7.44E-01 | 5.56E-01 |
| rs9806546 | rs8026932 | 1.98E+00 | 5.01E-01 |
| rs9806546 | rs6576602 | 6.52E-01 | 2.57E-01 |
| rs9806546 | rs890318 | 7.70E-01 | 6.63E-01 |
| rs9806546 | rs12593579 | 1.03E+00 | 9.45E-01 |
| rs9806546 | rs8038471 | 1.36E+00 | 3.36E-01 |
| rs9806546 | rs12905535 | 9.19E-01 | 8.17E-01 |
| rs9806546 | rs7165604 | 9.98E-01 | 9.98E-01 |
| rs9806546 | rs7174437 | 7.64E-01 | 7.23E-01 |
| rs9806546 | rs7178713 | 9.84E-01 | 9.63E-01 |
| rs9806546 | rs8026392 | 8.51E-01 | 6.36E-01 |
| rs9806546 | rs3212331 | 9.56E-01 | 8.99E-01 |
| rs8023959 | rs7179514 | 8.34E-01 | 6.87E-01 |
| rs8023959 | rs7171512 | 7.92E-01 | 6.19E-01 |
| rs8023959 | rs12442889 | 1.01E+00 | 9.84E-01 |
| rs8023959 | rs1367959 | 6.60E-01 | 2.31E-01 |
| rs8023959 | rs1863455 | 1.59E+00 | 4.34E-01 |
| rs8023959 | rs890317 | 1.28E+00 | 5.12E-01 |
| rs8023959 | rs11161329 | 1.40E+00 | 3.22E-01 |
| rs8023959 | rs12593482 | 1.36E+00 | 6.30E-01 |
| rs8023959 | rs1035751 | 1.45E+00 | 6.78E-01 |
| rs8023959 | rs7181473 | 9.54E-01 | 9.07E-01 |
| rs8023959 | rs17117279 | 8.19E-01 | 7.15E-01 |
| rs8023959 | rs1426224 | 1.39E+00 | 6.37E-01 |
| rs8023959 | rs1549482 | 1.04E+00 | 9.40E-01 |
| rs8023959 | rs4906896 | 2.11E+00 | 3.75E-02 |
| rs8023959 | rs737098 | 1.65E+00 | 2.12E-01 |
| rs8023959 | rs2315904 | 9.44E-01 | 9.20E-01 |
| rs8023959 | rs1863456 | 2.64E+00 | 5.95E-03 |
| rs8023959 | rs2162241 | 1.75E+00 | 9.65E-02 |
| rs8023959 | rs7180158 | 2.01E+00 | 1.41E-01 |
| rs8023959 | rs8026932 | 6.93E+00 | 4.68E-02 |
| rs8023959 | rs6576602 | 1.65E+00 | 1.86E-01 |
| rs8023959 | rs890318 | 9.06E-01 | 8.72E-01 |
| rs8023959 | rs12593579 | 2.74E+00 | 2.97E-02 |
| rs8023959 | rs8038471 | 4.82E-01 | 3.02E-02 |
| rs8023959 | rs12905535 | 1.17E+00 | 6.91E-01 |
| rs8023959 | rs7165604 | 1.72E+00 | 2.91E-01 |
| rs8023959 | rs7174437 | 1.46E+00 | 4.36E-01 |
| rs8023959 | rs7178713 | 1.69E+00 | 1.65E-01 |
| rs8023959 | rs8026392 | 2.06E+00 | 3.98E-02 |
| rs8023959 | rs3212331 | 9.14E-01 | 8.16E-01 |
| rs7179514 | rs7171512 | 8.67E-01 | 5.31E-01 |
| rs7179514 | rs12442889 | 1.27E+00 | 3.53E-01 |
| rs7179514 | rs1367959 | 7.02E-01 | 1.34E-01 |
| rs7179514 | rs1863455 | 1.26E+00 | 5.26E-01 |
| rs7179514 | rs890317 | 9.24E-01 | 7.56E-01 |
| rs7179514 | rs11161329 | 9.13E-01 | 6.92E-01 |
| rs7179514 | rs12593482 | 9.64E-01 | 9.49E-01 |
| rs7179514 | rs1035751 | 1.40E+00 | 3.97E-01 |
| rs7179514 | rs7181473 | 1.34E+00 | 2.12E-01 |
| rs7179514 | rs17117279 | 1.57E+00 | 1.60E-01 |
| rs7179514 | rs1426224 | 7.61E-01 | 6.32E-01 |
| rs7179514 | rs1549482 | 1.21E+00 | 5.07E-01 |
| rs7179514 | rs4906896 | 7.35E-01 | 1.62E-01 |
| rs7179514 | rs737098 | 7.43E-01 | 2.47E-01 |
| rs7179514 | rs2315904 | 1.26E+00 | 5.28E-01 |
| rs7179514 | rs1863456 | 6.70E-01 | 6.94E-02 |
| rs7179514 | rs2162241 | 7.30E-01 | 1.65E-01 |
| rs7179514 | rs7180158 | 1.01E+00 | 9.71E-01 |
| rs7179514 | rs8026932 | 3.06E-01 | 1.46E-01 |
| rs7179514 | rs6576602 | 1.01E+00 | 9.58E-01 |
| rs7179514 | rs890318 | 1.03E+00 | 9.33E-01 |
| rs7179514 | rs12593579 | 8.05E-01 | 4.61E-01 |
| rs7179514 | rs8038471 | 1.35E+00 | 1.77E-01 |
| rs7179514 | rs12905535 | 8.44E-01 | 4.96E-01 |
| rs7179514 | rs7165604 | 7.57E-01 | 4.59E-01 |
| rs7179514 | rs7174437 | 6.46E-01 | 2.82E-01 |
| rs7179514 | rs7178713 | 7.43E-01 | 2.15E-01 |
| rs7179514 | rs8026392 | 6.39E-01 | 4.64E-02 |
| rs7179514 | rs3212331 | 1.10E+00 | 7.17E-01 |
| rs7171512 | rs12442889 | 1.44E+00 | 1.72E-01 |
| rs7171512 | rs1367959 | 8.61E-01 | 5.20E-01 |
| rs7171512 | rs1863455 | 9.12E-01 | 7.97E-01 |
| rs7171512 | rs890317 | 8.32E-01 | 5.16E-01 |
| rs7171512 | rs11161329 | 7.70E-01 | 2.80E-01 |
| rs7171512 | rs12593482 | 8.22E-01 | 7.60E-01 |
| rs7171512 | rs1035751 | 1.37E+00 | 4.42E-01 |
| rs7171512 | rs7181473 | 1.12E+00 | 6.71E-01 |
| rs7171512 | rs17117279 | 9.62E-01 | 9.03E-01 |
| rs7171512 | rs1426224 | 7.43E-01 | 6.38E-01 |
| rs7171512 | rs1549482 | 1.23E+00 | 4.76E-01 |
| rs7171512 | rs4906896 | 1.06E+00 | 7.95E-01 |
| rs7171512 | rs737098 | 1.04E+00 | 8.86E-01 |
| rs7171512 | rs2315904 | 1.05E+00 | 9.03E-01 |
| rs7171512 | rs1863456 | 7.20E-01 | 1.53E-01 |
| rs7171512 | rs2162241 | 1.02E+00 | 9.35E-01 |
| rs7171512 | rs7180158 | 6.73E-01 | 2.07E-01 |
| rs7171512 | rs8026932 | 1.29E+00 | 7.01E-01 |
| rs7171512 | rs6576602 | 6.12E-01 | 6.84E-02 |
| rs7171512 | rs890318 | 7.50E-01 | 5.03E-01 |
| rs7171512 | rs12593579 | 6.30E-01 | 1.06E-01 |
| rs7171512 | rs8038471 | 1.56E+00 | 5.97E-02 |
| rs7171512 | rs12905535 | 1.14E+00 | 6.19E-01 |
| rs7171512 | rs7165604 | 6.73E-01 | 2.81E-01 |
| rs7171512 | rs7174437 | 7.39E-01 | 4.60E-01 |
| rs7171512 | rs7178713 | 1.26E+00 | 3.68E-01 |
| rs7171512 | rs8026392 | 8.77E-01 | 5.94E-01 |
| rs7171512 | rs3212331 | 1.21E+00 | 4.77E-01 |
| rs12442889 | rs1367959 | 8.51E-01 | 6.27E-01 |
| rs12442889 | rs1863455 | 5.52E-01 | 2.61E-01 |
| rs12442889 | rs890317 | 1.19E+00 | 6.22E-01 |
| rs12442889 | rs11161329 | 9.48E-01 | 8.58E-01 |
| rs12442889 | rs12593482 | 3.43E+00 | 1.18E-01 |
| rs12442889 | rs1035751 | 1.02E+00 | 9.69E-01 |
| rs12442889 | rs7181473 | 1.00E+00 | 9.91E-01 |
| rs12442889 | rs17117279 | 1.63E+00 | 1.90E-01 |
| rs12442889 | rs1426224 | 7.74E-01 | 7.48E-01 |
| rs12442889 | rs1549482 | 6.41E-01 | 1.48E-01 |
| rs12442889 | rs4906896 | 1.01E+00 | 9.73E-01 |
| rs12442889 | rs737098 | 1.14E+00 | 6.32E-01 |
| rs12442889 | rs2315904 | 1.62E+00 | 2.12E-01 |
| rs12442889 | rs1863456 | 1.02E+00 | 9.39E-01 |
| rs12442889 | rs2162241 | 1.16E+00 | 5.56E-01 |
| rs12442889 | rs7180158 | 8.80E-01 | 6.96E-01 |
| rs12442889 | rs8026932 | 3.54E-01 | 1.70E-01 |
| rs12442889 | rs6576602 | 9.86E-01 | 9.57E-01 |
| rs12442889 | rs890318 | 1.96E+00 | 1.32E-01 |
| rs12442889 | rs12593579 | 6.69E-01 | 1.86E-01 |
| rs12442889 | rs8038471 | 9.48E-01 | 8.25E-01 |
| rs12442889 | rs12905535 | 1.30E+00 | 3.36E-01 |
| rs12442889 | rs7165604 | 9.69E-01 | 9.34E-01 |
| rs12442889 | rs7174437 | 8.15E-01 | 6.21E-01 |
| rs12442889 | rs7178713 | 1.10E+00 | 7.15E-01 |
| rs12442889 | rs8026392 | 1.42E+00 | 1.69E-01 |
| rs12442889 | rs3212331 | 1.05E+00 | 8.53E-01 |
| rs1367959 | rs1863455 | 1.97E+00 | 1.78E-01 |
| rs1367959 | rs890317 | 1.29E+00 | 4.63E-01 |
| rs1367959 | rs11161329 | 1.69E+00 | 8.27E-02 |
| rs1367959 | rs12593482 | 1.20E-09 | 9.98E-01 |
| rs1367959 | rs1035751 | 1.30E+00 | 6.12E-01 |
| rs1367959 | rs7181473 | 1.47E+00 | 1.62E-01 |
| rs1367959 | rs17117279 | 1.35E+00 | 4.04E-01 |
| rs1367959 | rs1426224 | 8.02E-01 | 7.78E-01 |
| rs1367959 | rs1549482 | 1.48E+00 | 2.34E-01 |
| rs1367959 | rs4906896 | 1.50E+00 | 9.80E-02 |
| rs1367959 | rs737098 | 1.09E+00 | 7.59E-01 |
| rs1367959 | rs2315904 | 6.10E-01 | 2.47E-01 |
| rs1367959 | rs1863456 | 1.10E+00 | 6.87E-01 |
| rs1367959 | rs2162241 | 8.90E-01 | 6.49E-01 |
| rs1367959 | rs7180158 | 1.38E+00 | 3.01E-01 |
| rs1367959 | rs8026932 | 1.06E+00 | 9.43E-01 |
| rs1367959 | rs6576602 | 1.06E+00 | 8.31E-01 |
| rs1367959 | rs890318 | 4.64E-01 | 1.22E-01 |
| rs1367959 | rs12593579 | 1.48E+00 | 1.84E-01 |
| rs1367959 | rs8038471 | 7.86E-01 | 3.19E-01 |
| rs1367959 | rs12905535 | 1.22E+00 | 4.85E-01 |
| rs1367959 | rs7165604 | 1.39E+00 | 3.65E-01 |
| rs1367959 | rs7174437 | 1.56E+00 | 2.74E-01 |
| rs1367959 | rs7178713 | 1.22E+00 | 4.87E-01 |
| rs1367959 | rs8026392 | 1.48E+00 | 1.25E-01 |
| rs1367959 | rs3212331 | 7.76E-01 | 3.45E-01 |
| rs1863455 | rs890317 | 1.27E+00 | 6.25E-01 |
| rs1863455 | rs11161329 | 1.27E+00 | 5.89E-01 |
| rs1863455 | rs12593482 | 2.01E+00 | 5.92E-01 |
| rs1863455 | rs1035751 | 4.83E-01 | 5.14E-01 |
| rs1863455 | rs7181473 | 1.20E+00 | 6.25E-01 |
| rs1863455 | rs17117279 | 1.66E+00 | 3.74E-01 |
| rs1863455 | rs1426224 | 8.93E-01 | 8.69E-01 |
| rs1863455 | rs1549482 | 7.81E-01 | 5.55E-01 |
| rs1863455 | rs4906896 | 8.79E-01 | 7.37E-01 |
| rs1863455 | rs737098 | 8.00E-01 | 5.85E-01 |
| rs1863455 | rs2315904 | 3.36E-01 | 2.80E-01 |
| rs1863455 | rs1863456 | 6.41E-01 | 2.20E-01 |
| rs1863455 | rs2162241 | 6.44E-01 | 2.75E-01 |
| rs1863455 | rs7180158 | 9.43E-01 | 9.07E-01 |
| rs1863455 | rs8026932 | 7.44E-01 | 8.10E-01 |
| rs1863455 | rs6576602 | 7.33E-01 | 4.89E-01 |
| rs1863455 | rs890318 | 3.75E-01 | 3.47E-01 |
| rs1863455 | rs12593579 | 8.55E-01 | 7.27E-01 |
| rs1863455 | rs8038471 | 1.55E+00 | 2.36E-01 |
| rs1863455 | rs12905535 | 8.64E-01 | 7.32E-01 |
| rs1863455 | rs7165604 | 3.38E-01 | 1.72E-01 |
| rs1863455 | rs7174437 | 4.75E-01 | 3.54E-01 |
| rs1863455 | rs7178713 | 9.56E-01 | 9.10E-01 |
| rs1863455 | rs8026392 | 5.23E-01 | 1.08E-01 |
| rs1863455 | rs3212331 | 6.52E-01 | 3.66E-01 |
| rs890317 | rs11161329 | 5.81E-01 | 8.69E-02 |
| rs890317 | rs12593482 | 1.36E+00 | 7.14E-01 |
| rs890317 | rs1035751 | 9.48E-01 | 9.25E-01 |
| rs890317 | rs7181473 | 8.28E-01 | 4.82E-01 |
| rs890317 | rs17117279 | 4.17E-01 | 2.57E-02 |
| rs890317 | rs1426224 | 2.16E+00 | 1.88E-01 |
| rs890317 | rs1549482 | 1.50E+00 | 2.57E-01 |
| rs890317 | rs4906896 | 7.15E-01 | 1.96E-01 |
| rs890317 | rs737098 | 8.48E-01 | 5.70E-01 |
| rs890317 | rs2315904 | 1.46E+00 | 4.15E-01 |
| rs890317 | rs1863456 | 1.01E+00 | 9.59E-01 |
| rs890317 | rs2162241 | 1.18E+00 | 5.38E-01 |
| rs890317 | rs7180158 | 6.90E-01 | 3.08E-01 |
| rs890317 | rs8026932 | 2.82E+00 | 1.15E-01 |
| rs890317 | rs6576602 | 9.75E-01 | 9.34E-01 |
| rs890317 | rs890318 | 1.51E+00 | 4.10E-01 |
| rs890317 | rs12593579 | 9.59E-01 | 8.90E-01 |
| rs890317 | rs8038471 | 1.25E+00 | 4.03E-01 |
| rs890317 | rs12905535 | 6.17E-01 | 1.07E-01 |
| rs890317 | rs7165604 | 7.94E-01 | 6.32E-01 |
| rs890317 | rs7174437 | 7.88E-01 | 6.46E-01 |
| rs890317 | rs7178713 | 7.48E-01 | 3.15E-01 |
| rs890317 | rs8026392 | 6.15E-01 | 7.64E-02 |
| rs890317 | rs3212331 | 1.37E+00 | 2.43E-01 |
| rs11161329 | rs12593482 | 1.23E+00 | 7.84E-01 |
| rs11161329 | rs1035751 | 5.92E-01 | 3.45E-01 |
| rs11161329 | rs7181473 | 8.81E-01 | 5.96E-01 |
| rs11161329 | rs17117279 | 5.97E-01 | 1.35E-01 |
| rs11161329 | rs1426224 | 1.77E+00 | 4.49E-01 |
| rs11161329 | rs1549482 | 1.19E+00 | 5.49E-01 |
| rs11161329 | rs4906896 | 8.27E-01 | 4.12E-01 |
| rs11161329 | rs737098 | 8.74E-01 | 6.07E-01 |
| rs11161329 | rs2315904 | 1.06E+00 | 8.98E-01 |
| rs11161329 | rs1863456 | 9.29E-01 | 7.45E-01 |
| rs11161329 | rs2162241 | 1.02E+00 | 9.47E-01 |
| rs11161329 | rs7180158 | 8.06E-01 | 4.85E-01 |
| rs11161329 | rs8026932 | 2.30E+00 | 1.98E-01 |
| rs11161329 | rs6576602 | 9.55E-01 | 8.60E-01 |
| rs11161329 | rs890318 | 1.02E+00 | 9.58E-01 |
| rs11161329 | rs12593579 | 9.63E-01 | 8.90E-01 |
| rs11161329 | rs8038471 | 1.25E+00 | 3.22E-01 |
| rs11161329 | rs12905535 | 6.81E-01 | 1.51E-01 |
| rs11161329 | rs7165604 | 5.59E-01 | 1.79E-01 |
| rs11161329 | rs7174437 | 6.44E-01 | 3.31E-01 |
| rs11161329 | rs7178713 | 8.23E-01 | 4.51E-01 |
| rs11161329 | rs8026392 | 5.90E-01 | 2.98E-02 |
| rs11161329 | rs3212331 | 1.19E+00 | 4.94E-01 |
| rs12593482 | rs1035751 | 1.16E+00 | 8.48E-01 |
| rs12593482 | rs7181473 | 1.06E+00 | 9.21E-01 |
| rs12593482 | rs17117279 | 5.26E-01 | 5.80E-01 |
| rs12593482 | rs1426224 | 7.52E-01 | 8.17E-01 |
| rs12593482 | rs1549482 | 1.10E+00 | 8.86E-01 |
| rs12593482 | rs4906896 | 5.48E-01 | 3.07E-01 |
| rs12593482 | rs737098 | 6.08E-01 | 4.79E-01 |
| rs12593482 | rs2315904 | 8.88E-01 | 9.21E-01 |
| rs12593482 | rs1863456 | 6.07E-01 | 3.85E-01 |
| rs12593482 | rs2162241 | 7.95E-01 | 7.06E-01 |
| rs12593482 | rs7180158 | 5.75E-01 | 4.54E-01 |
| rs12593482 | rs8026932 | 1.95E+00 | 4.40E-01 |
| rs12593482 | rs6576602 | 5.86E-01 | 3.84E-01 |
| rs12593482 | rs890318 | 7.12E-01 | 7.52E-01 |
| rs12593482 | rs12593579 | 9.23E-01 | 8.92E-01 |
| rs12593482 | rs8038471 | 1.47E+00 | 5.03E-01 |
| rs12593482 | rs12905535 | 6.48E-01 | 5.16E-01 |
| rs12593482 | rs7165604 | 6.11E-01 | 5.60E-01 |
| rs12593482 | rs7174437 | 7.45E-10 | 9.99E-01 |
| rs12593482 | rs7178713 | 9.08E-01 | 8.74E-01 |
| rs12593482 | rs8026392 | 4.74E-01 | 2.11E-01 |
| rs12593482 | rs3212331 | 1.81E+00 | 2.27E-01 |
| rs1035751 | rs7181473 | 3.38E-01 | 1.14E-01 |
| rs1035751 | rs17117279 | 7.80E-01 | 7.68E-01 |
| rs1035751 | rs1426224 | 2.24E-09 | 9.99E-01 |
| rs1035751 | rs1549482 | 2.58E-01 | 2.04E-01 |
| rs1035751 | rs4906896 | 1.04E+00 | 9.29E-01 |
| rs1035751 | rs737098 | 1.45E+00 | 4.13E-01 |
| rs1035751 | rs2315904 | 1.11E+00 | 8.60E-01 |
| rs1035751 | rs1863456 | 1.00E+00 | 9.93E-01 |
| rs1035751 | rs2162241 | 1.33E+00 | 5.17E-01 |
| rs1035751 | rs7180158 | 4.84E-01 | 2.04E-01 |
| rs1035751 | rs8026932 | 7.59E-01 | 8.24E-01 |
| rs1035751 | rs6576602 | 7.30E-01 | 4.39E-01 |
| rs1035751 | rs890318 | 1.35E+00 | 6.26E-01 |
| rs1035751 | rs12593579 | 6.42E-01 | 3.74E-01 |
| rs1035751 | rs8038471 | 9.03E-01 | 7.99E-01 |
| rs1035751 | rs12905535 | 1.40E+00 | 4.49E-01 |
| rs1035751 | rs7165604 | 6.67E-01 | 5.07E-01 |
| rs1035751 | rs7174437 | 7.18E-01 | 5.94E-01 |
| rs1035751 | rs7178713 | 1.51E+00 | 3.48E-01 |
| rs1035751 | rs8026392 | 1.25E+00 | 5.90E-01 |
| rs1035751 | rs3212331 | 1.77E+00 | 2.34E-01 |
| rs7181473 | rs17117279 | 1.05E+00 | 9.11E-01 |
| rs7181473 | rs1426224 | 4.76E-01 | 2.07E-01 |
| rs7181473 | rs1549482 | 6.59E-01 | 2.28E-01 |
| rs7181473 | rs4906896 | 1.02E+00 | 9.27E-01 |
| rs7181473 | rs737098 | 7.94E-01 | 4.17E-01 |
| rs7181473 | rs2315904 | 9.76E-01 | 9.42E-01 |
| rs7181473 | rs1863456 | 7.88E-01 | 3.10E-01 |
| rs7181473 | rs2162241 | 8.28E-01 | 4.57E-01 |
| rs7181473 | rs7180158 | 7.87E-01 | 4.77E-01 |
| rs7181473 | rs8026932 | 1.68E+00 | 4.98E-01 |
| rs7181473 | rs6576602 | 7.95E-01 | 3.89E-01 |
| rs7181473 | rs890318 | 9.00E-01 | 7.88E-01 |
| rs7181473 | rs12593579 | 8.99E-01 | 7.34E-01 |
| rs7181473 | rs8038471 | 1.30E+00 | 2.71E-01 |
| rs7181473 | rs12905535 | 6.70E-01 | 1.61E-01 |
| rs7181473 | rs7165604 | 4.76E-01 | 8.23E-02 |
| rs7181473 | rs7174437 | 6.40E-01 | 3.45E-01 |
| rs7181473 | rs7178713 | 7.34E-01 | 2.55E-01 |
| rs7181473 | rs8026392 | 6.93E-01 | 1.45E-01 |
| rs7181473 | rs3212331 | 1.07E+00 | 7.83E-01 |
| rs17117279 | rs1426224 | 6.04E-01 | 6.64E-01 |
| rs17117279 | rs1549482 | 1.35E+00 | 5.48E-01 |
| rs17117279 | rs4906896 | 6.50E-01 | 1.79E-01 |
| rs17117279 | rs737098 | 7.02E-01 | 3.72E-01 |
| rs17117279 | rs2315904 | 1.56E+00 | 3.80E-01 |
| rs17117279 | rs1863456 | 7.84E-01 | 4.48E-01 |
| rs17117279 | rs2162241 | 1.02E+00 | 9.45E-01 |
| rs17117279 | rs7180158 | 6.98E-01 | 4.72E-01 |
| rs17117279 | rs8026932 | 2.08E+00 | 3.35E-01 |
| rs17117279 | rs6576602 | 8.77E-01 | 7.40E-01 |
| rs17117279 | rs890318 | 1.45E+00 | 5.20E-01 |
| rs17117279 | rs12593579 | 8.23E-01 | 6.44E-01 |
| rs17117279 | rs8038471 | 1.44E+00 | 2.53E-01 |
| rs17117279 | rs12905535 | 5.07E-01 | 8.81E-02 |
| rs17117279 | rs7165604 | 3.85E-01 | 1.32E-01 |
| rs17117279 | rs7174437 | 4.18E-01 | 2.17E-01 |
| rs17117279 | rs7178713 | 6.01E-01 | 1.71E-01 |
| rs17117279 | rs8026392 | 5.18E-01 | 6.76E-02 |
| rs17117279 | rs3212331 | 1.16E+00 | 6.32E-01 |
| rs1426224 | rs1549482 | 2.56E-01 | 2.27E-01 |
| rs1426224 | rs4906896 | 1.67E+00 | 3.94E-01 |
| rs1426224 | rs737098 | 1.79E+00 | 3.13E-01 |
| rs1426224 | rs2315904 | 2.48E-09 | 9.99E-01 |
| rs1426224 | rs1863456 | 1.00E+00 | 9.97E-01 |
| rs1426224 | rs2162241 | 1.22E+00 | 7.14E-01 |
| rs1426224 | rs7180158 | 1.02E+00 | 9.86E-01 |
| rs1426224 | rs8026932 | 1.59E-09 | 9.99E-01 |
| rs1426224 | rs6576602 | 7.19E-01 | 7.15E-01 |
| rs1426224 | rs890318 | 2.63E-09 | 9.99E-01 |
| rs1426224 | rs12593579 | 8.57E-01 | 8.69E-01 |
| rs1426224 | rs8038471 | 1.03E+00 | 9.50E-01 |
| rs1426224 | rs12905535 | 1.73E+00 | 3.46E-01 |
| rs1426224 | rs7165604 | 5.06E-01 | 5.82E-01 |
| rs1426224 | rs7174437 | 2.22E+00 | 5.91E-01 |
| rs1426224 | rs7178713 | 1.14E+00 | 8.15E-01 |
| rs1426224 | rs8026392 | 9.15E-01 | 8.80E-01 |
| rs1426224 | rs3212331 | 5.35E-01 | 4.73E-01 |
| rs1549482 | rs4906896 | 1.35E+00 | 3.03E-01 |
| rs1549482 | rs737098 | 1.12E+00 | 7.42E-01 |
| rs1549482 | rs2315904 | 7.90E-01 | 5.40E-01 |
| rs1549482 | rs1863456 | 9.19E-01 | 7.53E-01 |
| rs1549482 | rs2162241 | 9.11E-01 | 7.61E-01 |
| rs1549482 | rs7180158 | 7.12E-01 | 3.84E-01 |
| rs1549482 | rs8026932 | 8.97E-01 | 9.02E-01 |
| rs1549482 | rs6576602 | 7.14E-01 | 2.53E-01 |
| rs1549482 | rs890318 | 7.39E-01 | 5.06E-01 |
| rs1549482 | rs12593579 | 8.26E-01 | 5.91E-01 |
| rs1549482 | rs8038471 | 1.02E+00 | 9.43E-01 |
| rs1549482 | rs12905535 | 1.07E+00 | 8.36E-01 |
| rs1549482 | rs7165604 | 5.58E-01 | 2.15E-01 |
| rs1549482 | rs7174437 | 7.89E-01 | 6.47E-01 |
| rs1549482 | rs7178713 | 1.22E+00 | 5.43E-01 |
| rs1549482 | rs8026392 | 1.06E+00 | 8.55E-01 |
| rs1549482 | rs3212331 | 1.07E+00 | 8.34E-01 |
| rs4906896 | rs737098 | 6.83E-01 | 1.64E-01 |
| rs4906896 | rs2315904 | 2.22E+00 | 4.52E-02 |
| rs4906896 | rs1863456 | 8.39E-01 | 4.16E-01 |
| rs4906896 | rs2162241 | 8.46E-01 | 4.78E-01 |
| rs4906896 | rs7180158 | 9.29E-01 | 8.34E-01 |
| rs4906896 | rs8026932 | 3.56E-01 | 1.18E-01 |
| rs4906896 | rs6576602 | 1.61E+00 | 8.40E-02 |
| rs4906896 | rs890318 | 2.29E+00 | 6.02E-02 |
| rs4906896 | rs12593579 | 6.99E-01 | 2.48E-01 |
| rs4906896 | rs8038471 | 1.21E+00 | 3.93E-01 |
| rs4906896 | rs12905535 | 7.93E-01 | 3.89E-01 |
| rs4906896 | rs7165604 | 8.69E-01 | 7.40E-01 |
| rs4906896 | rs7174437 | 1.03E+00 | 9.53E-01 |
| rs4906896 | rs7178713 | 6.36E-01 | 8.74E-02 |
| rs4906896 | rs8026392 | 8.90E-01 | 6.13E-01 |
| rs4906896 | rs3212331 | 1.23E+00 | 4.11E-01 |
| rs737098 | rs2315904 | 2.74E+00 | 5.98E-02 |
| rs737098 | rs1863456 | 1.02E+00 | 9.44E-01 |
| rs737098 | rs2162241 | 1.19E+00 | 5.44E-01 |
| rs737098 | rs7180158 | 9.56E-01 | 9.35E-01 |
| rs737098 | rs8026932 | 1.28E+00 | 8.42E-01 |
| rs737098 | rs6576602 | 1.34E+00 | 4.80E-01 |
| rs737098 | rs890318 | 2.77E+00 | 8.94E-02 |
| rs737098 | rs12593579 | 8.79E-01 | 8.09E-01 |
| rs737098 | rs8038471 | 1.21E+00 | 5.31E-01 |
| rs737098 | rs12905535 | 8.55E-01 | 6.08E-01 |
| rs737098 | rs7165604 | 6.71E-01 | 5.46E-01 |
| rs737098 | rs7174437 | 1.01E+00 | 9.91E-01 |
| rs737098 | rs7178713 | 8.22E-01 | 5.11E-01 |
| rs737098 | rs8026392 | 6.69E-01 | 1.97E-01 |
| rs737098 | rs3212331 | 2.00E+00 | 4.80E-02 |
| rs2315904 | rs1863456 | 1.70E+00 | 2.05E-01 |
| rs2315904 | rs2162241 | 1.66E+00 | 2.04E-01 |
| rs2315904 | rs7180158 | 1.59E+00 | 5.20E-01 |
| rs2315904 | rs8026932 | 9.71E-01 | 9.70E-01 |
| rs2315904 | rs6576602 | 6.60E-01 | 3.81E-01 |
| rs2315904 | rs890318 | 6.02E-01 | 5.79E-01 |
| rs2315904 | rs12593579 | 1.20E+00 | 7.28E-01 |
| rs2315904 | rs8038471 | 5.84E-01 | 2.00E-01 |
| rs2315904 | rs12905535 | 2.79E+00 | 3.22E-02 |
| rs2315904 | rs7165604 | 1.48E+00 | 6.45E-01 |
| rs2315904 | rs7174437 | 1.13E+00 | 9.01E-01 |
| rs2315904 | rs7178713 | 2.97E+00 | 1.73E-02 |
| rs2315904 | rs8026392 | 2.60E+00 | 1.85E-02 |
| rs2315904 | rs3212331 | 7.21E-01 | 4.38E-01 |
| rs1863456 | rs2162241 | 1.23E+00 | 4.02E-01 |
| rs1863456 | rs7180158 | 6.86E-01 | 3.42E-01 |
| rs1863456 | rs8026932 | 4.95E-01 | 4.14E-01 |
| rs1863456 | rs6576602 | 1.34E+00 | 3.51E-01 |
| rs1863456 | rs890318 | 3.32E+00 | 4.04E-02 |
| rs1863456 | rs12593579 | 6.44E-01 | 2.13E-01 |
| rs1863456 | rs8038471 | 9.04E-01 | 6.36E-01 |
| rs1863456 | rs12905535 | 1.11E+00 | 7.13E-01 |
| rs1863456 | rs7165604 | 7.56E-01 | 5.72E-01 |
| rs1863456 | rs7174437 | 1.14E+00 | 8.12E-01 |
| rs1863456 | rs7178713 | 1.01E+00 | 9.69E-01 |
| rs1863456 | rs8026392 | 1.19E+00 | 4.66E-01 |
| rs1863456 | rs3212331 | 1.40E+00 | 1.91E-01 |
| rs2162241 | rs7180158 | 7.37E-01 | 5.12E-01 |
| rs2162241 | rs8026932 | 2.98E+00 | 2.29E-01 |
| rs2162241 | rs6576602 | 8.12E-01 | 4.39E-01 |
| rs2162241 | rs890318 | 2.47E+00 | 9.16E-02 |
| rs2162241 | rs12593579 | 8.84E-01 | 7.09E-01 |
| rs2162241 | rs8038471 | 8.92E-01 | 6.48E-01 |
| rs2162241 | rs12905535 | 1.31E+00 | 3.22E-01 |
| rs2162241 | rs7165604 | 5.42E-01 | 2.80E-01 |
| rs2162241 | rs7174437 | 6.75E-01 | 5.92E-01 |
| rs2162241 | rs7178713 | 1.48E+00 | 1.15E-01 |
| rs2162241 | rs8026392 | 8.05E-01 | 3.57E-01 |
| rs2162241 | rs3212331 | 1.95E+00 | 1.05E-02 |
| rs7180158 | rs8026932 | 5.23E-10 | 9.98E-01 |
| rs7180158 | rs6576602 | 9.50E-01 | 8.92E-01 |
| rs7180158 | rs890318 | 1.58E+00 | 5.76E-01 |
| rs7180158 | rs12593579 | 6.72E-01 | 3.11E-01 |
| rs7180158 | rs8038471 | 1.44E+00 | 3.67E-01 |
| rs7180158 | rs12905535 | 1.02E+00 | 9.73E-01 |
| rs7180158 | rs7165604 | 1.30E+00 | 6.04E-01 |
| rs7180158 | rs7174437 | 1.46E+00 | 4.98E-01 |
| rs7180158 | rs7178713 | 4.94E-01 | 1.69E-01 |
| rs7180158 | rs8026392 | 1.49E+00 | 2.07E-01 |
| rs7180158 | rs3212331 | 6.48E-01 | 3.84E-01 |
| rs8026932 | rs6576602 | 3.19E-01 | 2.25E-01 |
| rs8026932 | rs890318 | 3.05E+00 | 2.97E-01 |
| rs8026932 | rs12593579 | 5.68E-10 | 9.98E-01 |
| rs8026932 | rs8038471 | 6.93E-01 | 6.62E-01 |
| rs8026932 | rs12905535 | 3.33E+00 | 2.49E-01 |
| rs8026932 | rs7165604 | 5.25E-10 | 9.99E-01 |
| rs8026932 | rs7174437 | 7.70E-10 | 9.99E-01 |
| rs8026932 | rs7178713 | 4.47E+00 | 8.67E-02 |
| rs8026932 | rs8026392 | 6.51E-01 | 6.49E-01 |
| rs8026932 | rs3212331 | 2.87E+00 | 1.56E-01 |
| rs6576602 | rs890318 | 1.21E+00 | 7.54E-01 |
| rs6576602 | rs12593579 | 1.13E+00 | 6.93E-01 |
| rs6576602 | rs8038471 | 7.41E-01 | 3.50E-01 |
| rs6576602 | rs12905535 | 1.39E+00 | 4.18E-01 |
| rs6576602 | rs7165604 | 1.34E+00 | 5.59E-01 |
| rs6576602 | rs7174437 | 1.29E+00 | 6.41E-01 |
| rs6576602 | rs7178713 | 1.11E+00 | 7.77E-01 |
| rs6576602 | rs8026392 | 2.34E+00 | 1.71E-03 |
| rs6576602 | rs3212331 | 5.40E-01 | 3.81E-02 |
| rs890318 | rs12593579 | 2.29E+00 | 2.23E-01 |
| rs890318 | rs8038471 | 3.07E-01 | 4.51E-02 |
| rs890318 | rs12905535 | 2.61E+00 | 8.88E-02 |
| rs890318 | rs7165604 | 1.44E+00 | 7.15E-01 |
| rs890318 | rs7174437 | 8.04E-01 | 8.58E-01 |
| rs890318 | rs7178713 | 3.86E+00 | 1.16E-02 |
| rs890318 | rs8026392 | 2.71E+00 | 6.26E-02 |
| rs890318 | rs3212331 | 1.07E+00 | 9.04E-01 |
| rs12593579 | rs8038471 | 1.11E+00 | 7.65E-01 |
| rs12593579 | rs12905535 | 1.14E+00 | 7.95E-01 |
| rs12593579 | rs7165604 | 9.01E-01 | 8.29E-01 |
| rs12593579 | rs7174437 | 1.15E+00 | 7.96E-01 |
| rs12593579 | rs7178713 | 7.96E-01 | 5.20E-01 |
| rs12593579 | rs8026392 | 1.39E+00 | 2.54E-01 |
| rs12593579 | rs3212331 | 9.64E-01 | 9.09E-01 |
| rs8038471 | rs12905535 | 1.14E+00 | 6.77E-01 |
| rs8038471 | rs7165604 | 1.15E+00 | 7.84E-01 |
| rs8038471 | rs7174437 | 8.65E-01 | 7.91E-01 |
| rs8038471 | rs7178713 | 1.18E+00 | 5.39E-01 |
| rs8038471 | rs8026392 | 9.72E-01 | 9.08E-01 |
| rs8038471 | rs3212331 | 7.53E-01 | 2.70E-01 |
| rs12905535 | rs7165604 | 8.12E-01 | 7.53E-01 |
| rs12905535 | rs7174437 | 1.20E+00 | 8.39E-01 |
| rs12905535 | rs7178713 | 7.85E-01 | 4.28E-01 |
| rs12905535 | rs8026392 | 7.16E-01 | 2.62E-01 |
| rs12905535 | rs3212331 | 1.75E+00 | 9.72E-02 |
| rs7165604 | rs7174437 | 1.17E+00 | 7.80E-01 |
| rs7165604 | rs7178713 | 3.46E-01 | 8.82E-02 |
| rs7165604 | rs8026392 | 1.40E+00 | 5.04E-01 |
| rs7165604 | rs3212331 | 6.96E-01 | 5.41E-01 |
| rs7174437 | rs7178713 | 4.29E-01 | 2.86E-01 |
| rs7174437 | rs8026392 | 2.80E+00 | 8.33E-02 |
| rs7174437 | rs3212331 | 5.16E-01 | 3.01E-01 |
| rs7178713 | rs8026392 | 5.55E-01 | 3.32E-02 |
| rs7178713 | rs3212331 | 2.28E+00 | 7.37E-03 |
| rs8026392 | rs3212331 | 1.62E+00 | 1.47E-01 |
