# Supplementary material for: Genetic variation in GABRB3 is associated with Asperger syndrome and multiple endophenotypes relevant to autism
Source: Mol Autism. 2013 Dec 9;4:48. doi: 10.1186/2040-2392-4-48 (PMC3903107; doi:10.1186/2040-2392-4-48)
Supplement: Additional file 4 — GABRB3 case–control (AS) results for haplotype analysis. AS, Asperger syndrome. [file 2040-2392-4-48-S4.docx]

**Additional File 3**

**GABRB3 case-control (AS) results for haplotype analysis**

| No of SNPs | Starting SNP | Ending SNP | Empirical P-value |
| --- | --- | --- | --- |
| 2 | rs12593579 | rs8038471 | **0.00582** |
| 2 | rs12905535 | rs7165604 | **0.01768** |
| 2 | rs7180158 | rs8026932 | **0.0304** |
| 2 | rs1863456 | rs2162241 | **0.03284** |
| 2 | rs6576602 | rs890318 | **0.03576** |
| 2 | rs2162241 | rs7180158 | **0.03882** |
| 2 | rs4906896 | rs737098 | 0.05188 |
| 2 | rs890318 | rs12593579 | 0.0904 |
| 2 | rs7165604 | rs7174437 | 0.1942 |
| 2 | rs8038471 | rs12905535 | 0.2708 |
| 2 | rs7174437 | rs8026392 | 0.3287 |
| 2 | rs737098 | rs2315904 | 0.6515 |
| 2 | rs8026932 | rs6576602 | 0.761 |
| 2 | rs1549482 | rs4906896 | 0.9169 |
| 2 | rs11636966 | rs9806546 | 0.9899 |
| 2 | rs1863455 | rs890317 | 0.9966 |
| 2 | rs8026392 | rs3212331 | 0.9979 |
| 2 | rs11161329 | rs12593482 | 0.9996 |
| 2 | rs10873636 | rs11636966 | 0.9999 |
| 2 | rs7183249 | rs2114485 | 1 |
| 2 | rs2114485 | rs11631940 | 1 |
| 2 | rs11631940 | rs1432007 | 1 |
| 2 | rs1432007 | rs1426217 | 1 |
| 2 | rs1426217 | rs10519563 | 1 |
| 2 | rs10519563 | rs12440905 | 1 |
| 2 | rs12440905 | rs17646555 | 1 |
| 2 | rs17646555 | rs12437672 | 1 |
| 2 | rs12437672 | rs1582760 | 1 |
| 2 | rs1582760 | rs2873027 | 1 |
| 2 | rs2873027 | rs12438141 | 1 |
| 2 | rs12438141 | rs10873636 | 1 |
| 2 | rs9806546 | rs8023959 | 1 |
| 2 | rs8023959 | rs7179514 | 1 |
| 2 | rs7179514 | rs7171512 | 1 |
| 2 | rs7171512 | rs12442889 | 1 |
| 2 | rs12442889 | rs1367959 | 1 |
| 2 | rs1367959 | rs1863455 | 1 |
| 2 | rs890317 | rs11161329 | 1 |
| 2 | rs12593482 | rs1035751 | 1 |
| 2 | rs1035751 | rs7181473 | 1 |
| 2 | rs7181473 | rs17117279 | 1 |
| 2 | rs17117279 | rs1426224 | 1 |
| 2 | rs1426224 | rs1549482 | 1 |
| 2 | rs2315904 | rs1863456 | 1 |
| 3 | rs12593579 | rs12905535 | **0.01192** |
| 3 | rs890318 | rs8038471 | **0.0145** |
| 3 | rs4906896 | rs2315904 | **0.02002** |
| 3 | rs8026932 | rs890318 | **0.0209** |
| 3 | rs1863456 | rs7180158 | **0.02438** |
| 3 | rs8038471 | rs7165604 | **0.03052** |
| 3 | rs6576602 | rs12593579 | 0.05312 |
| 3 | rs2162241 | rs8026932 | 0.0532 |
| 3 | rs12905535 | rs7174437 | 0.0587 |
| 3 | rs7180158 | rs6576602 | 0.07838 |
| 3 | rs7165604 | rs8026392 | 0.09304 |
| 3 | rs2315904 | rs2162241 | 0.1389 |
| 3 | rs1549482 | rs737098 | 0.3223 |
| 3 | rs7174437 | rs3212331 | 0.3971 |
| 3 | rs737098 | rs1863456 | 0.4415 |
| 3 | rs1426224 | rs4906896 | 0.97 |
| 3 | rs11161329 | rs1035751 | 0.9962 |
| 3 | rs890317 | rs12593482 | 0.9992 |
| 3 | rs11631940 | rs1426217 | 0.9997 |
| 3 | rs1035751 | rs17117279 | 0.9997 |
| 3 | rs10873636 | rs9806546 | 0.9999 |
| 3 | rs11636966 | rs8023959 | 0.9999 |
| 3 | rs1367959 | rs890317 | 0.9999 |
| 3 | rs7183249 | rs11631940 | 1 |
| 3 | rs2114485 | rs1432007 | 1 |
| 3 | rs1432007 | rs10519563 | 1 |
| 3 | rs1426217 | rs12440905 | 1 |
| 3 | rs10519563 | rs17646555 | 1 |
| 3 | rs12440905 | rs12437672 | 1 |
| 3 | rs17646555 | rs1582760 | 1 |
| 3 | rs12437672 | rs2873027 | 1 |
| 3 | rs1582760 | rs12438141 | 1 |
| 3 | rs2873027 | rs10873636 | 1 |
| 3 | rs12438141 | rs11636966 | 1 |
| 3 | rs9806546 | rs7179514 | 1 |
| 3 | rs8023959 | rs7171512 | 1 |
| 3 | rs7179514 | rs12442889 | 1 |
| 3 | rs7171512 | rs1367959 | 1 |
| 3 | rs12442889 | rs1863455 | 1 |
| 3 | rs1863455 | rs11161329 | 1 |
| 3 | rs12593482 | rs7181473 | 1 |
| 3 | rs7181473 | rs1426224 | 1 |
| 3 | rs17117279 | rs1549482 | 1 |
| 4 | rs890318 | rs12905535 | **0.01002** |
| 4 | rs6576602 | rs8038471 | **0.02428** |
| 4 | rs7180158 | rs890318 | **0.03832** |
| 4 | rs12593579 | rs7165604 | **0.0454** |
| 4 | rs8026932 | rs12593579 | **0.04774** |
| 4 | rs1863456 | rs8026932 | 0.05146 |
| 4 | rs2162241 | rs6576602 | 0.06888 |
| 4 | rs8038471 | rs7174437 | 0.1262 |
| 4 | rs4906896 | rs1863456 | 0.1308 |
| 4 | rs2315904 | rs7180158 | 0.1553 |
| 4 | rs7165604 | rs3212331 | 0.1884 |
| 4 | rs12905535 | rs8026392 | 0.2013 |
| 4 | rs737098 | rs2162241 | 0.2409 |
| 4 | rs1549482 | rs2315904 | 0.3687 |
| 4 | rs1426224 | rs737098 | 0.6324 |
| 4 | rs17117279 | rs4906896 | 0.9956 |
| 4 | rs1863455 | rs12593482 | 0.9965 |
| 4 | rs11161329 | rs7181473 | 0.9994 |
| 4 | rs11631940 | rs10519563 | 0.9995 |
| 4 | rs890317 | rs1035751 | 0.9997 |
| 4 | rs1432007 | rs12440905 | 0.9998 |
| 4 | rs10873636 | rs8023959 | 0.9999 |
| 4 | rs7183249 | rs1432007 | 1 |
| 4 | rs2114485 | rs1426217 | 1 |
| 4 | rs1426217 | rs17646555 | 1 |
| 4 | rs10519563 | rs12437672 | 1 |
| 4 | rs12440905 | rs1582760 | 1 |
| 4 | rs17646555 | rs2873027 | 1 |
| 4 | rs12437672 | rs12438141 | 1 |
| 4 | rs1582760 | rs10873636 | 1 |
| 4 | rs2873027 | rs11636966 | 1 |
| 4 | rs12438141 | rs9806546 | 1 |
| 4 | rs11636966 | rs7179514 | 1 |
| 4 | rs9806546 | rs7171512 | 1 |
| 4 | rs8023959 | rs12442889 | 1 |
| 4 | rs7179514 | rs1367959 | 1 |
| 4 | rs7171512 | rs1863455 | 1 |
| 4 | rs12442889 | rs890317 | 1 |
| 4 | rs1367959 | rs11161329 | 1 |
| 4 | rs12593482 | rs17117279 | 1 |
| 4 | rs1035751 | rs1426224 | 1 |
| 4 | rs7181473 | rs1549482 | 1 |

Significant P-values are written in bold.
